# Multi-domain human-oriented approach to evaluate human comfort in outdoor environments

**DOI:** 10.1007/s00484-022-02338-7

**Published:** 2022-08-09

**Authors:** Roberta Jacoby Cureau, Ilaria Pigliautile, Ioannis Kousis, Anna Laura Pisello

**Affiliations:** 1 CIRIAF – Interuniversity Research Center on Pollution and Environment Mauro Felli – University of Perugia, 06125 Perugia, Italy; 2 Department of Engineering, University of Perugia, Via G. Duranti 93, 06125 Perugia, Italy

**Keywords:** Human comfort, Multi-domain, Multi-disciplinary, Environmental monitoring, Urban parks

## Abstract

Human comfort outdoors is widely investigated, but most studies explore the comfort domains singularly. This paper aimed to evaluate human comfort in parks, verifying the importance of using a multi-domain (simultaneously evaluating thermal, visual, acoustic, and air quality) and multi-disciplinary (combining environmental and social fields) approach. A walk through a pre-defined path from one park to another was repeated twice per day on four consecutive days in June, with three participants per walk. The two investigated parks are in central Italy and were chosen because they differ in their design and spatial characteristics. Environmental data were recorded with an innovative wearable device during the whole walk, and surveys were used to assess people’s perceptions of the parks. Despite observed differences in collected physical parameters, the survey’s responses were similar, and different comfort domains showed dependence on each other in the two parks. Logistic regression models were developed for each park, and they revealed that the qualitative information predicted the overall comfort level more accurately than the environmental data. In detail, the models based on environmental data resulted in *R*^*2*^ equal to 0.126 and 0.111 in Parks 1 and 2, respectively, whereas using the survey answers increased it up to 0.820 (Park 1) and 0.806 (Park 2). This study contributes to addressing the gap in multi-domain comfort studies outdoors and confirms the importance of using multi-disciplinary and multi-domain approaches for a complete comfort analysis, supporting holistic human-biometeorology-oriented models and forecasting opportunities that can promote improvements in urban environmental quality and liveability.

**Supplementary Information:**

The online version contains supplementary material available at 10.1007/s00484-022-02338-7.

## Introduction

According to the United Nations, about 55% of the world’s population lives in cities, and this number is expected to increase to 68% by 2050 (United Nations Human Settlements Programme 2020). The rapid growth of urbanization intensified the discussions related to the environmental quality outdoors as this process is negatively affecting urban environments bringing consequences that harm the liveability of cities (Peckens et al. 2018; Peng et al. 2019).

Outdoor environments are an extension of living spaces, being places for several activities and social interactions that have the potential to affect people’s health and well-being positively and contribute to urban liveability and vitality (Chen and Ng, 2012; Lau and Choi, 2021; Peng et al., 2019). That is why human comfort outdoors is widely investigated while considering human-biometeorological conditions (Giannaros et al. 2018) and with a special focus on cities environment, where thermal bioclimate may provide key insight for the management of urban design (Rodríguez Algeciras and Matzarakis 2016; Rodríguez Algeciras et al. 2016). The usual approach for studying outdoor comfort is to evaluate just one domain of comfort at a time. Some studies demonstrated how air temperature, mean radiant temperature, and wind speed affect thermal sensation (Peng et al. 2019; Zhang et al. 2021). Jamei and Rajagopalan (2019) investigated the effects of street design on physiological equivalent temperature, air temperature, and mean radiant temperature. Air quality is usually explored in terms of people’s exposure to air pollutants (Pantavou et al. 2018; Pigliautile et al. 2020; Samad and Vogt 2021). Regarding acoustics, soundscape (the acoustic environment as perceived by a person in context) research has been growing recently (Aletta et al. 2019), with several studies using this approach to evaluate acoustic comfort in urban environments (Zhao et al. 2018; Mascolo et al. 2020; Mancini et al. 2021; Jo and Jeon 2021a; Yang et al. 2022). Concerning the visual domain, outdoor glare and over lighting are typically associated with visual discomfort (Brotas and Wienold 2019; Pan and Du 2021).

Whereas people are exposed to all environmental factors simultaneously, there are only a few studies investigating multi-domain comfort (Kousis and Pisello, 2020), and they are mostly related to indoors (Geng et al. 2017; Yang et al. 2018; Jamrozik et al. 2018; Chinazzo et al. 2019; Yang and Moon 2019). According to Torresin et al. (2018), different comfort domains (i.e., thermal, acoustic, visual, and air quality) are interconnected and thus should be investigated under the same conceptual framework.

Studies investigating interactions and crossed effects of comfort domains in outdoor environments are still few. Lam et al. (2020) investigated the association between visual and thermal domains on three university campuses and concluded that improving visual comfort could simultaneously enhance outdoor thermal comfort perception. Lau and Choi (2021) studied the relations between perceived acoustics and thermal comfort in urban areas and showed that thermal sensation had a negative association with acoustics vote. D’Alessandro et al. (2018) identified that people’s perception of the acoustic and overall environment was affected by climate conditions in the external area of a university. Engel et al. (2018) found correlations between background sound quality and subjects’ air quality perception in urban parks and busy streets. Even if these studies indicate that comfort domains are interrelated, this approach is still underexplored in outdoor environments and should be further addressed to reach a full understanding of human comfort.

Furthermore, factors other than the environment by itself influence human comfort. It is already well-reported that physiological, psychological, and cultural aspects can affect human comfort perception (D’Oca et al. 2018; Castaldo et al. 2018). In the outdoors, it has been identified that positive and negative emotions influence thermal sensation (Zhang et al. 2021). Ma et al. (2021) concluded that pedestrian comfort is affected by environmental factors but also by people’s subjective perceptions, namely built environment satisfaction, thermal sensation, perceived air quality, and perceived loudness. Other studies also related that air quality perception is influenced by personal factors, such as age, gender, health symptoms, and smoke status (Pantavou et al. 2017, 2018). Therefore, the human dimension should be implemented in comfort experiments combined with environmental monitoring, which has been done by using social science methods, like surveys and interviews (D’Alessandro et al. 2018; Lam et al. 2020; Liu et al. 2021; Ma et al. 2021; Mancini et al. 2021; Pantavou et al. 2018; Peng et al. 2019), and physiological measurements (Chokhachian et al. 2018; Niu et al. 2020).

In general, parks are key spots in urban spaces as they provide environmental and social benefits, promoting physical activities practice, reducing stress, and boosting social connections (Jiang et al. 2019). Moreover, these places reduce air pollution (Fares et al. 2020) and mitigate urban microclimate (Pioppi et al. 2021). The cooling effects of urban parks have been extensively studied (Lu et al. 2017; Yan et al. 2018; Aram et al. 2020; Li et al. 2020). Furthermore, urban greenery also contributes to noise attenuation (Cohen et al. 2014). Jo and Jeon (2021a) studied the visual and acoustic perception in urban parks and concluded that greenery and water elements improve acoustic and visual satisfaction. Therefore, parks place a key role in cities, as they are usually more comfortable than the urban surrounding in terms of all comfort domains.

In the same way that urban landscapes change over time according to the urbanization process (Weng 2007), urban parks also vary. In the past, an urban park was a focus of nature inside a city. Today, they should be more attractive and serve several purposes to increase their entertaining capacity (Matovnikov and Matovnikova 2016). In fact, environmental design affects comfort directly because of its effect on the physical metrics (Jamei and Rajagopalan 2019), but there is also a non-direct influence on the comfort level perceived by people. Satisfaction with aesthetic quality was found to be related to thermal perception in outdoor environments (Lau and Choi 2021), and the percentage of natural and other visual features in a place can influence the perception of acoustic and overall comfort (D’Alessandro et al. 2018).

Under this background, given the lack of comfort studies investigating the relation between comfort domains outdoors, this paper presents a procedure to evaluate human comfort in outdoor environments using a multi-domain and multi-disciplinary approach. The aim of the analysis was to verify the relations between different comfort domains and the need to involve more than one discipline in comfort studies outdoors. Key multi-domain parameters related to air quality, thermal, acoustic, and visual domains were measured with a novel wearable device developed for environmental monitoring on a hyperlocal scale from a pedestrian perspective. Surveys were used to assess human comfort perception. The method was applied in two urban parks, as these places generally provide more comfortable conditions than the urban surrounding in all the domains according to the literature. These two parks were chosen because of their different designs, one traditional and the other more innovative, to verify if this contrast affects human comfort perception and the relations between comfort domains.

## Materials and methods

This study reports on the outdoor evaluation of pedestrians’ comfort. Under this framework, a walking path was devised to investigate people’s perceptions of thermal, visual, and acoustic conditions while simultaneously monitoring the main environmental parameters representative of the four domains. The route was specifically planned to start and finish in two different parks to evaluate their comfort level in these places and verify whether their divergent designs could affect the environmental parameters and people’s perceptions.

### Case studies

**Fig. 1:**
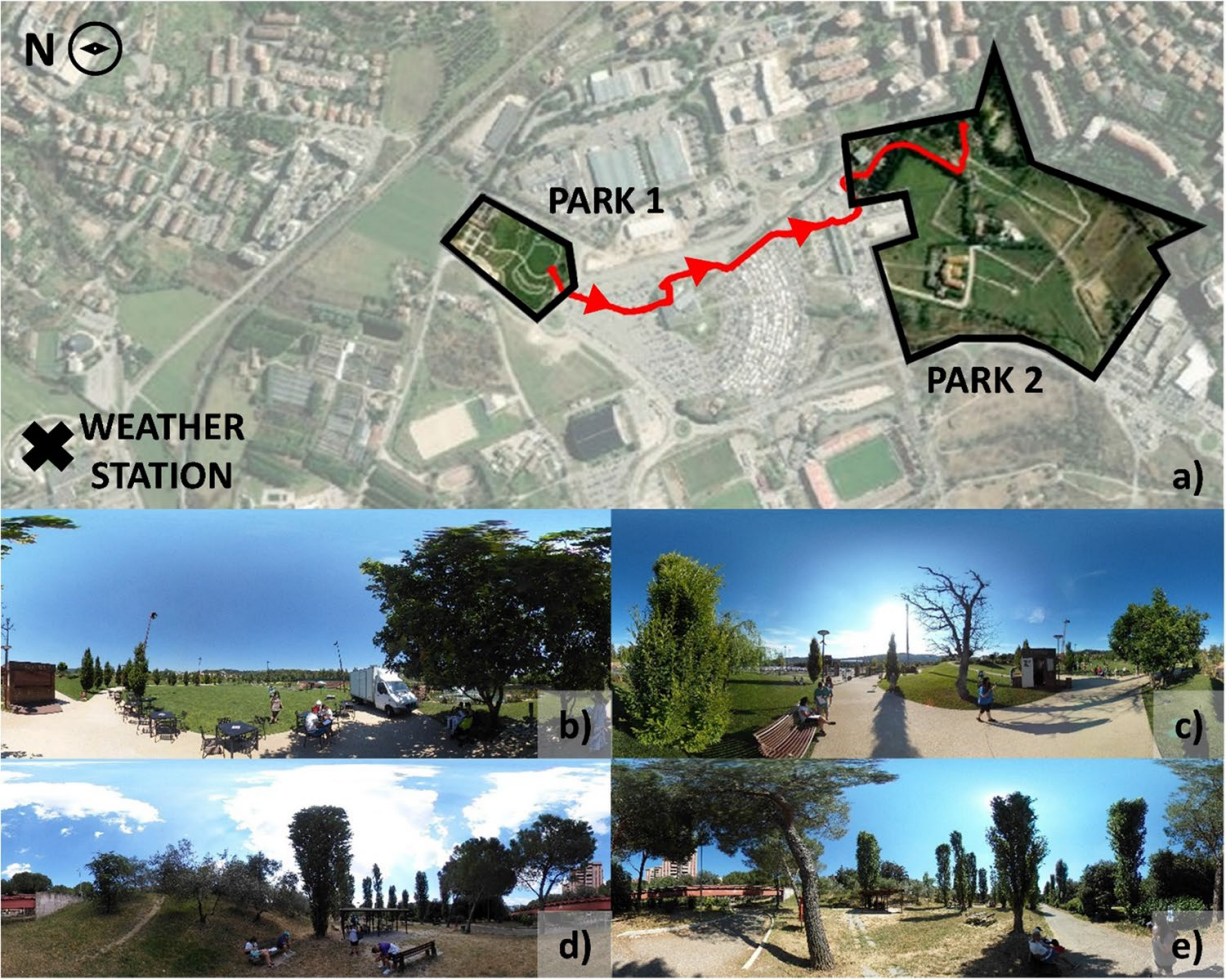
**a** Location of the parks and the weather station; (**b**, **c**) Park 1; (**d**, **e**) Park 2

The two parks under investigation are located in Perugia, central Italy, characterized by a humid subtropical climate (climate zone Cfa) according to the Köppen-Geiger classification (Kottek et al. 2006). They are approximately 1 km apart. Figure 1a shows their location on a map. The red line shows the path taken on the walks, and the arrows indicate the direction this path was completed. The figure also presents the position of the weather station used as a reference. Figure 1b and c show the specific position of the participants in Park 1 and Fig. 1d and e in Park 2.

The experiment was conducted for 4 days in June 2021 and always started at Park 1. It is the most recent among the two analyzed parks, has an area of 3 hectares, and is managed by a private company. The green area is integrated with sidewalks, seats, a lake with a fountain, a playground for children in the middle of the park, an open arena, and a restaurant. The entire park has music in the background all the time. Park 1 is relatively new, has more activity options, and serves several purposes, following a more innovative park design (Matovnikov and Matovnikova 2016).

Park 2 is larger than Park 1 (around 17 hectares) but hosts a smaller number of functionalities compared to the other and presents fewer urban features. There is a large green area with some sidewalks and seats, and it has a playground for children. This park is crossed by the elevated tracks of a public transportation system, which is a noise source that can be heard at some places in the park.

### The experiment

The experiment consisted of a walk from one park to another following a pre-defined path two times a day, at 2 pm and at 6 pm local time (that is, Central European Summer Time), for four consecutive days, from June 12^th^ to 15^th^. The two times were selected for being symmetric to the hour with maximum air temperature (usually around 4 pm (Pioppi et al. 2020; Labdaoui et al. 2021)). Hence, the first walk of the day took place while the heat level was increasing and the other while it was reducing. Each walk was approximately 1 km and lasted around 55 min. A smart wearable monitoring station was specifically designed on a backpack as a light wearable device (Cureau et al. 2022) to record environmental data during the whole procedure. One person carried the wearable during the entire monitoring, always staying close to the participants. The walks were always completed in the same direction, i.e., Park 1 was always the starting point, and Park 2 the ending point.

In each walk, three volunteers took part in the experiment for a total of 24 subjects. Those people were contacted some days before the experiment, consented to provide their information, and were informed about the general framework of the research (evaluation of environmental perception in urban outdoors) without giving them many details to avoid influencing their responses. They were also aware that they could leave the experiment anytime they wanted. Finally, they were informed that all the provided data would be treated anonymously according to the General Data Protection Regulation (GDPR) 2018. The limited number of participants per route was due to the COVID-19 pandemic contingencies. Even if the activity took place outdoors, bigger groups would not respect the ban on the gathering (i.e., groups of five persons at maximum). Besides the walking period, participants spent a minimum of 10 min in each park, as quiet as possible, to acclimatize with the environment. They were free to stay where they preferred, within a radius of a few meters from the monitoring equipment. After the acclimatization period, participants answered a survey on their environmental perception, including comfort, sensation, and preference evaluation on a 5-point scale for thermal, visual, and acoustic domains. For the whole walk duration (about 75 min), some people who were already in the parks (here named as local people) were also asked to respond to a similar survey to increase the number of answers and improve the analysis.

### Environmental data

**Fig. 2:**
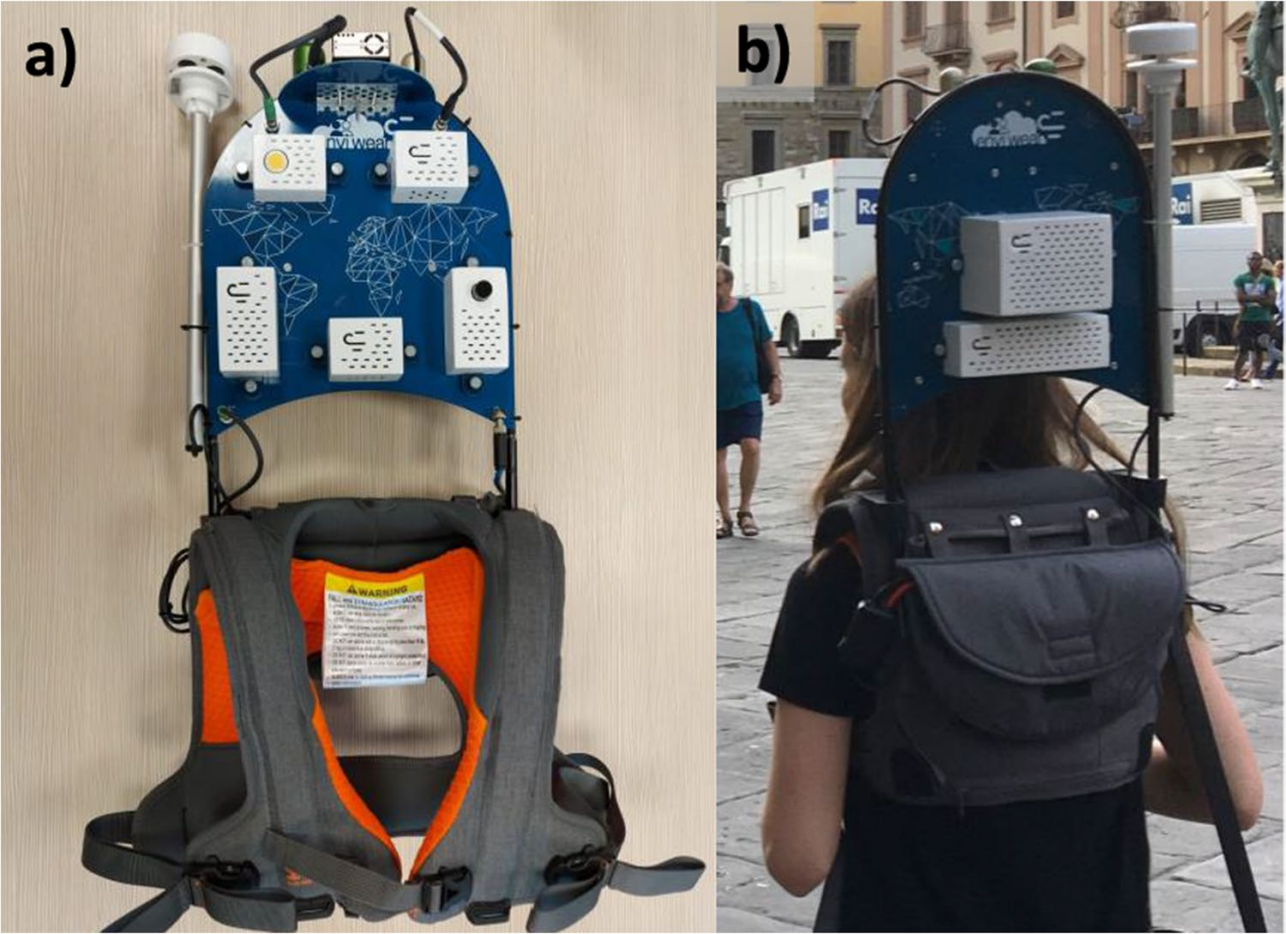
The wearable equipment: (**a**) front view; (**b**) in use

A wearable device equipped with several calibrated sensors was used in this experiment (Cureau et al. 2022). It monitors the geographic position using a GPS, air temperature, relative humidity, wind velocity and direction, pressure, global solar radiation, illuminance, and CO_2_ and particulate matter concentration (PM_1.0_, PM_2.5_, and PM_10_). This novel equipment was developed to measure environmental data outdoors on a hyperlocal scale from a pedestrian perspective. The advantage of this device is that it allows checking fine-grain spatial variability of the parameters resulting in a spatial resolution that cannot be achieved by fixed weather station networks. Its compact design also allows it to reach points that monitoring systems based on cars cannot. Figure 2 shows the wearable equipment. More details about the device and its sensors are found in Cureau et al. (2022).

To characterize the parks in terms of acoustics, sound pressure level was measured with a digital sonometer (model C.A. 832) while the participants were waiting to respond to the survey. Additionally, data from a fixed weather station were got to evaluate the effect of time on temperature and relative humidity measurements made by the wearable in the two parks. The temporal effects were assessed by comparing the variations of the measured parameters at the beginning and the end of each walk by the weather station and by the wearable equipment. This weather station is located approximately 1 km far from Park 1 and 1.7 km from Park 2 and monitors air temperature, relative humidity, solar radiation, and wind speed.

### Multi-domain comfort surveys

To assess variations in comfort perception and the relations between comfort domains, qualitative data were also collected. Two different surveys were designed to investigate people’s assessment of the urban park’s environments, distinguishing between the group of (i) environmental walk participants and (ii) people spending their time in the specific areas of investigation (local people). The former group was specifically pre-defined before the walk, while the latter was randomly assessed during the walk.

The survey applied to the participants was divided into two parts. The first one focused on personal information (gender, age, height, weight, and worn garments) and was answered only at the beginning of the experiment.

The second part was related to multi-domain comfort and was based on the recommendations of the ISO 10551:2019 (International Organization for Standardization 2019) and the Method A of the ISO/TS 12913–2:2018 (International Organization for Standardization 2018). This part was filled twice, one at each park. Regarding the thermal and visual domains, they were asked about comfort, acceptability, sensation, and preference. Wind sensation and preference and humidity sensation and preference were also asked, based on the questionnaire applied by Lam et al. (2021). Concerning the acoustics, participants answered about noise perception, acoustic quality perception, and whether they consider the sound environment appropriate to the place. They also answered about their overall comfort status. All the questions used a 5-point scale measurement (Likert-like scale (Bavaresco et al. 2020) for the responses related to comfort, acceptability, sensation, preference, and perception). In the end, participants were asked if they were directly exposed to the sun.

The survey was very similar for local people, excluding the questions about their height, weight, and clothing. The complete surveys are presented in Appendix A.

### Data analysis

Statistical methods were applied to analyze the data collected through the wearable device and the surveys. Descriptive statistics, hypothesis tests, and multinomial logistic regression were the main techniques used. This analysis was performed using the free software R (R Core Team 2021), using the nnet (Venables and Ripley 2002), DescTools (Signorell 2017), and car (Fox and Weisberg 2019) packages. The significance level adopted in this study was 5% for all the tests.

Environmental data for each park in every walk were compared using the Mann–Whitney test. This method is used to compare two independent samples (Corder and Foreman 2011), and its application aimed to verify if the parks in the same walk differed in some environmental parameters. A non-parametric approach was chosen because most samples did not follow the normal distribution (McKnight and Najab 2010).

Similarly, votes from the surveys were also evaluated using the Mann–Whitney test to compare the parks in terms of differences in answers to the same question. Only the responses from local people were considered in this analysis because these samples are not paired and are bigger than the participants’ samples.

Survey answers for different domains were compared to see whether one comfort domain affects the others. This analysis was performed using Fisher’s exact test (Hoffman 2015). Each park was evaluated separately to verify if the distinctions between their design influence the multi-domain comfort perception. This test considered both participants’ and local people’s responses.

Finally, multinomial logistic regression models were developed to analyze which type of information better predicts people’s overall comfort level (dependent variable). This method generalizes the conventional logistic regression when the dependent variable is categorical with more than two levels (Psomas et al. 2021). In this case, the dependent value is the overall comfort vote (5-point scale). Six models were developed for each park, using different independent variables (demographic information, monitored environmental data, and varied combinations of qualitative data obtained through the comfort survey) to verify which one better predicts the overall comfort vote. More information about these models is provided in Appendix D.

Models from the same park were evaluated and compared using the McFadden pseudo *R*^2^. The pseudo *R*^2^ varies between 0 and 1, and the higher the value, the better the model fit (Domencich and McFadden 1975). The chi-square test was used to check whether the independent variables included in the model were significant or not to predict people’s overall comfort level.

## Results

### Microclimate boundary conditions

Parameters measured with the fixed weather station during the entire 4 days of the experiment (June 12^th^, 13^th^, 14^th^, and 15^th^) are presented in Fig. B1 in Supplementary Material. The 4 days were similar in terms of air temperature, relative humidity, and wind speed. The sky was clearer on the 13^th^ and 15^th^ than on the other two days. Analyzing only the periods when the walks were carried out, at 2 pm, the temperature increased in time, while relative humidity decreased (the only exception was the relative humidity measured on the 15^th^ at 2 pm). Inverse profiles were observed for both temperature and humidity during the 6 pm walks. Solar radiation was decreasing in time for both periods on all days, despite the 12^th^ and 14^th^ at 2 pm, when no tendency was detected, probably because there were a few clouds in the sky on these days. No trend was observed for wind speed during the walks.

The environmental data measured with the wearable equipment and the digital sonometer within the two parks in each walk are presented, respectively, in Fig. B2 and B3 in Supplementary Material. Due to a technical issue that occurred with the wearable device during the walk on the 14^th^ at 6 pm, the environmental data of this walk are not available.

In general, the temperature was similar to or higher in Park 2, and wind speed was higher in Park 1. The heating and cooling trends of air temperature along the walks, observed with the fixed weather station at 2 pm and 6 pm, respectively, were not identified in all walks with the wearable equipment. Park 1 area is more open, while in Park 2 there are more barriers that can limit the wind field (Fig. 1b–e), which can explain this distinction in the wind speed in the two parks. Relative humidity was more variable in Park 1 than in Park 2. The last day of experiments was drier than the others on both walks.

While PM_2.5_ and PM_10_ concentrations were almost the same within both parks, CO_2_ concentration was higher in Park 2. Mean PM_2.5_ and PM_10_ concentrations did not exceed the 24-h mean guideline established by the World Health Organization (2021) (15 µg/m^3^ for PM_2.5_ and 45 µg/m^3^ for PM_10_) in any walk. PM_2.5_ reached the limit a few times on the 14^th^ at 2 pm but did not exceed it.

Mean solar radiation and illuminance varied a lot among all the walks. Solar radiation and illuminance ranges were larger in Park 2. Park 2 has more trees (Fig. 1d and e), which increases the availability of shaded areas.

The sound pressure level was always higher (average difference of 10 dB) in Park 1. According to an Italian Decree that establishes limit values of sound level emissions (Decree of the President of the Council of Ministers 1997), parks should have a maximum sound level of 50 dB during the day. Both parks exceed this limit on all walks.

Observed differences in air temperature and relative humidity measured with the weather station during the walk periods are presented in Table C1 in Supplementary Material. The table also shows the difference between the mean values measured in Parks 1 and 2 (starting and ending points of the route, respectively) with the wearable equipment. The negative signs indicate that the parameter values decreased in time during the route. The heating and cooling patterns identified with the weather station were not always seen with the wearable equipment. Moreover, at 2 pm, the differences observed with the wearable are higher than the ones related to the weather station in almost all the walks, while at 6 pm, the highest ranges are associated with the weather station. There is a time effect on these two parameters, but the divergent differences indicate that the spatial characteristics of these two parks have a bigger impact on temperature and relative humidity than time, considering the interval of the walks (about 75 min).

The differences between the two parks in terms of environmental parameters, presented in Fig. B2 and B3, were also tested for their statistical significance using the Mann–Whitney test. The test was repeated 63 times (nine environmental parameters and seven walks of experiments with data available). In 76% of them, the results confirm that the differences between parks were significant (5% significance level). Sound level pressure and wind speed were significantly different in all walks. These results suggest that the two parks are relatively different concerning the environmental parameters measured.

### Surveys

A total of 24 people (three subjects in each walk) were involved in the experiments as participants. Participants directly took part in the walk and answered the survey in both parks. They were mostly female (63%), between 19 and 62 years old (mean 32.7 years and standard deviation 15.4 years). Local people (volunteers who were already in the parks and anonymously answered the survey) were 127 in Park 1 and 93 in Park 2. They were 50% male and 50% female, ranging from 16 to 77 years old (mean 37.4 years and standard deviation 16.2 years). Merging participants and locals, the mean age was 36.9 years old (standard deviation 16.1 years), with 52% females. Separating the whole group according to their age group, 50% of them were young (less than 35 years old), 43% were middle-aged (between 35 and 65 years old), and 7% were elderly (more than 65 years old).

Fig. B4, B5, B6, and B7 in Supplementary Material summarize the answers from the survey for both participants and local people in each park under different domains, i.e., thermal (Fig. B4 and B5), visual (Fig. B6), and acoustic (Fig. B7). The bars show the percentage of votes for each option, and the lines represent the mean vote for the question. The answers are separated by the time of the walk.

For questions related to the thermal domain, people generally voted for “hot” in thermal sensation in Park 1 (62% of votes at 2 pm and 68% at 6 pm, Fig. B4a), but declared that the thermal environment is “acceptable” or “clearly acceptable” (69% of votes at 2 pm and 80% at 6 pm for these two options, Fig. B4d). In Park 2, the thermal sensation was also predominantly “hot” (63% of votes at 2 pm and 70% at 6 pm), but acceptability was mostly “neutral” and “acceptable” (56% of votes at 2 pm and 62% at 6 pm to these two options). In Park 1, thermal preference (Fig. B4b) was predominantly for “no changes” (67% of votes at 2 pm and 72% at 6 pm). On the other hand, in Park 2, at 2 pm, votes were predominantly for “colder” (63%), and at 6 pm, they were divided between “colder” and “no changes” (more than 45% of votes for each option). This difference may result from the highest average air temperature in Park 2 and the highest wind speed in Park 1 on most walks. Thermal comfort votes differed among time (Fig. B4c): at 2 pm, the votes were distributed among “neutral” and “comfortable” (mean vote equals 0.4 in Park 1 and 0.3 in Park 2); at 6 pm, they were mainly for “comfortable” (more than 50% of votes in both parks) even for Park 2, where people usually preferred “neutral” and “colder”. Votes for wind sensation and preference (Fig. B5a and B5b) were similar for both parks, with most votes for “light wind” (sensation) and “no changes” (preference) in both parks and periods, even though the wind speed was higher in Park 1. Most people voted for “neutral” in humidity sensation, i.e., mean votes of 0.3 (Park 1) and 0.4 (Park 2) at 2 pm, and 0.1 (Park 1) and 0.3 (Park 2) at 6 pm (Fig. B5c). Humidity preference votes were distributed among “less humid” and “no changes” (Fig. B5d). In general, the relative humidity was similar in the two parks during the same walk, usually a little higher in Park 1. However, the mean vote for humidity sensation was slightly higher in Park 2, where the wind speed monitored and perceived is lower. Then, humidity perception could have been affected by the wind perception.

Concerning the visual domain (Fig. B6), despite the differences in illuminance between the two parks (Fig. B2h), people voted similarly: the majority answered for “bright” (sensation), “no changes” (preference), “comfortable” (comfort), and “clearly acceptable” (acceptability). This result suggests that in outdoor environments, visual discomfort is caused by factors other than illuminance, like glare, for example, and this should be better explored in future studies.

In terms of acoustics, Park 1 was perceived as noisier than Park 2 (mean vote equals 0.2 in Park 1 in both periods, against − 0.2 at 2 pm and 0.1 at 6 pm in Park 2, Fig. B7a), which is in line with the highest average sound pressure level at this place. However, at 6 pm, acoustic quality (Fig. B7b) was better evaluated in Park 1 (mean vote of 0.8 in Park 1 against 0.6 in Park 2). It indicates that variables other than sound pressure, like the sound spectrum, for example, probably influence acoustic comfort since a noisier environment was not always associated with inferior acoustic quality. It is important to note that both parks exceeded the sound level limit established by the Italian legislation for this kind of place, but even though they were well evaluated in terms of acoustic quality, with a positive mean vote at 2 pm and 6 pm. The judgments regarding whether the acoustic environment is adequate for the place (Fig. B7c) were better in Park 2 (mean vote equals 2.9 in Park 2 in both periods against 2.5 and 2.6 in Park 1 at 2 and 6 pm, respectively).

Around 60% of people declared that their overall comfort level was “comfortable” in Park 1 at 2 pm and 6 pm and in Park 2 at 6 pm (Fig. B7d). In Park 2 at 2 pm, votes were divided between “neutral” (43%) and “comfortable” (37%), as it was for thermal comfort, suggesting a stronger relation between this domain and general comfort when compared to visual and acoustics.

Survey answers were also compared considering different age groups (young, middle-aged, and elderly). No significant differences were observed between young and middle age while elderly people differed on some questions. In detail, they have more votes for “very hot” in thermal sensation (28% of votes), and “windy” in wind sensation (50%) than the other groups (respectively, 6% and 22% of votes). Regarding the visual domain, even though the negative votes represent a small amount of the total in the questions related to comfort (3%) and acceptability (4%), they were all done by middle-aged and young people. All elderly voted for “no changes” in visual preference. On the contrary, 12% of middle-aged and young people voted for a preference different from “no changes”. Concerning acoustics, no elderly voted in the extremes for noise perception (“very silent” or “very noisy”), and all their votes were neutral or positive for acoustic quality perception. In contrast, among the other groups, 20% of the votes were for “very silent”, 20% for “very noisy”, and 7% gave a negative evaluation of the acoustic quality perception. These differences can be due to varied environmental perceptions in people of this age group. However, the elderly are the category with the few people interviewed (only 18 people), and these results may not represent the entire population.

Answers from all the questions in the survey were compared using the Mann–Whitney test to see if people responded differently in the two parks during the same walk. The test was repeated 120 times (15 questions and eight walks), and only 14% of the data provide convincing evidence to declare that the answers were different between the two parks. In contrast, the differences among environmental data were significant in 76% of the tests. This suggests that environmental data may not be the only variable influencing human comfort and environmental perception.

A multi-domain investigation was performed to test whether there was a relation between answers for questions related to different domains. The *p* values for Fisher’s exact test are presented in Table C2 in Supplementary Material. In general, comfort domains were more related to each other in Park 1 than in Park 2. In Park 1, all variables were related to at least two others. The variables with more influence in other domains were thermal acceptability, visual comfort, and visual acceptability. In Park 2, thermal sensation, visual preference, and noise perception did not present a dependence relation with any other variable.

The more evident dependence between comfort domains observed in Park 1 than in Park 2 was confirmed by applying this test for the overall comfort question. In Park 1, only the noise perception was not related to overall comfort (*p* = 0.101). Conversely, in Park 2, thermal sensation (*p* = 0.075), visual sensation (*p* = 0.168), visual perception (*p* = 0.160), and any question about acoustics (noise perception *p* = 0.291, acoustic quality *p* = 0.083, and acoustic environment appropriate to the place *p* = 0.419) were not related to the general comfort level.

**Fig. 3:**
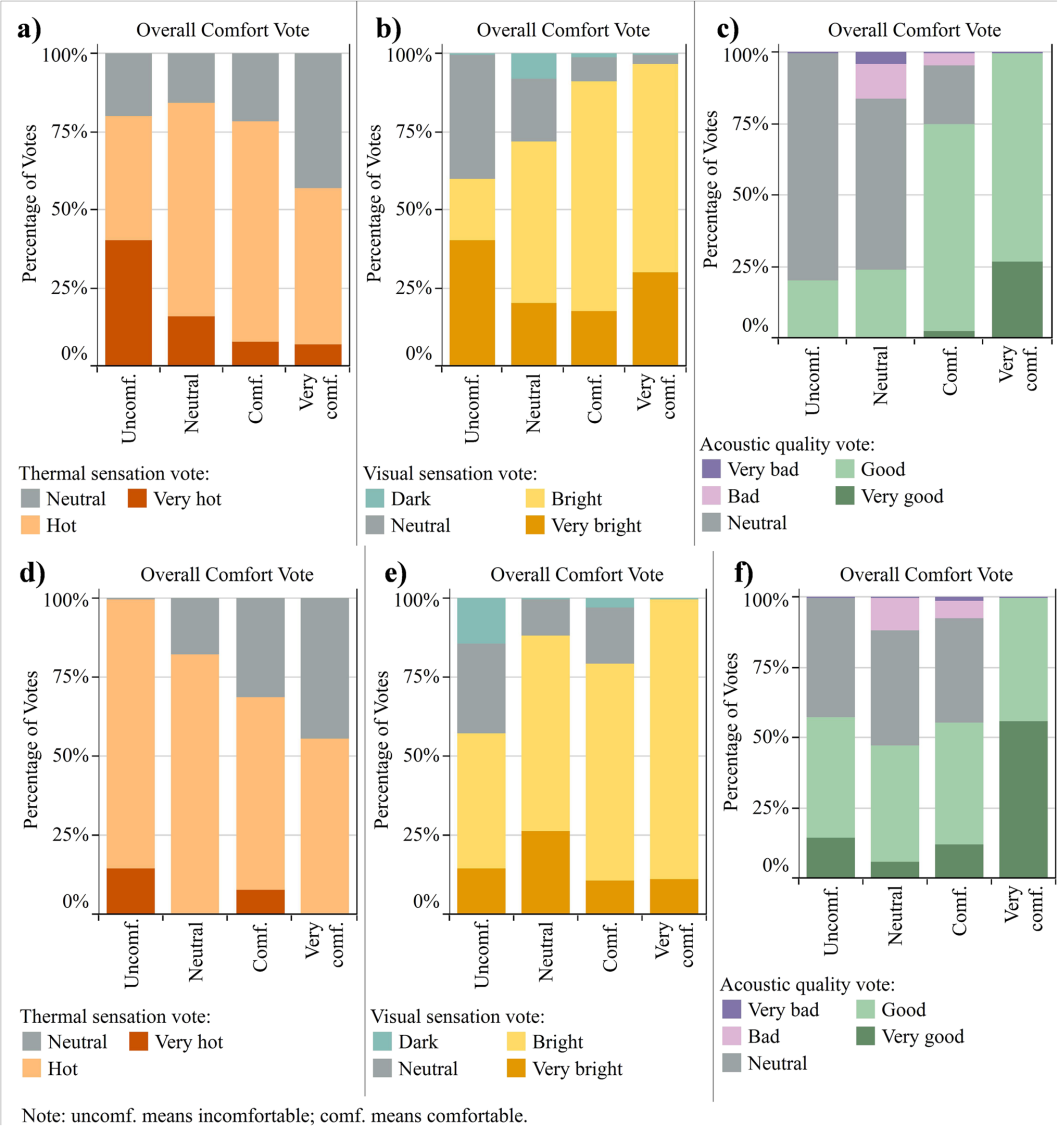
Percentage for overall comfort vote according to (**a**) thermal sensation in Park 1; (**b**) visual sensation in Park 1; (**c**) acoustic quality in Park 1; (**d**) thermal sensation in Park 2; (**e**) visual sensation in Park 2; and (**f**) acoustic quality in Park 2

Figure 3 presents how the overall comfort level is related to the responses concerning thermal sensation, visual sensation, and acoustic quality for Parks 1 and 2. In Fig. 3a, b, and 3c, the variation in votes among different overall comfort categories suggests that the overall comfort vote depends on these variables in Park 1. This relation was also observed by the Fisher’s exact test that resulted in *p* values lower than 0.05 (thermal sensation *p* = 0.045, visual sensation *p* = 0.016, and acoustic quality *p* < 0.001). The differences are less notable in Park 2 (Fig. 3d, 3e, and 3f), as confirmed by *p* values higher than 0.05 when applying Fisher’s exact test.

It is important to note that the two parks did not present a major trigger of discomfort, which is also proved by the surveys’ responses. Mean comfort and acceptability votes are all positive. Therefore, the dependence between specific domains found in this analysis could be different in other circumstances with particular sources of discomfort.

### Models to predict the overall comfort level

Six multinomial logistic regression models were developed for every park, each one with different information as independent variables to evaluate which better predicts people’s overall comfort level. Appendix D presents the outcomes for all logistic regression models.

Demographic information and environmental data did not provide good results in any park. The best model for both parks was the one that used thermal, visual, and acoustic questions simultaneously (*R*^2^ = 0.820 in Park 1 and *R*^2^ = 0.806 in Park 2), highlighting the existence of a combined effect between domains on overall comfort perception. The models based on the qualitative assessment of each domain assumed individually provided higher prediction accuracy to those using environmental data, which again supports the hypothesis that factors other than environmental parameters also affect overall comfort. In Park 1, the model based on acoustic questions (*R*^2^ = 0.328) was slightly better in overall comfort prediction than the ones based on thermal and visual assessments, while in Park 2, thermal responses provided a more suitable result (*R*^2^ = 0.445). Indeed, from the surveys’ answers, there is also evidence that the thermal domain has a stronger influence than the others on overall comfort perception in Park 2.

The analysis of variables’ significance for the models provided different results in the two parks. In Park 1, no variable was significant (5% significance level) in models 1 and 2. In model 3, only thermal comfort was significant (*p* = 0.001); in model 4, visual comfort (*p* = 0.002) and visual acceptability (*p* = 0.006); and in model 5, acoustic quality (*p* < 0.001) and whether the acoustic environment is appropriate to the place (*p* = 0.006). In Park 2, models 1, 2, 4, and 5 did not present any significant variables for predicting overall comfort. These are also the models with the worst fit. In model 3, thermal acceptability was significant (*p* = 0.010).

Model 6 fitted the best in both parks, but the significance of the variables was different between them, similarly to the other models. In Park 1, almost all questions were significant, with exception of visual sensation (*p* = 0.838) and noise perception (*p* = 0.197). These variables were also not significant for models 4 (only visual questions) and 5 (only acoustic questions) for this park. In contrast, in model 6 for Park 2, only thermal comfort (*p* = 0.005) was significant.

## Discussions

Parks are important places within the urban context as other studies have already demonstrated that their characteristics are associated with better conditions regarding thermal, visual, acoustic, and air quality domains (Cohen et al. 2014; Fares et al. 2020; Pioppi et al. 2021; Jo and Jeon 2021b). The two investigated parks showed to be different in terms of physical parameters registered when comparing them through statistical tests. Moreover, the comparison between data collected by the wearable device and the fixed weather station showed that the spatial characteristics of these two parks have a bigger effect on temperature and relative humidity than the time during the walk periods. The heating and cooling patterns observed with the fixed weather station were not identified with the wearable device in all walks, which is another indicator that spatial boundaries had a larger influence on air temperature and relative humidity than time. Furthermore, this demonstrates the capability of these parks to mitigate the urban microclimate within their areas. This mitigation effect of parks was already confirmed by other studies (Lu et al. 2017; Yan et al. 2018; Aram et al. 2020; Li et al. 2020).

It is also remarkable that both parks exceeded the sound level limits established by the Italian legislation, and despite that, they were well evaluated in terms of acoustic quality. Indeed, Park 1, which was perceived as noisier (further proved by the sound pressure level measurements), was also better assessed during the 6 pm walks. This suggests that acoustic comfort is not only related to sound pressure level, and other variables, such as the sound spectrum, should also be considered. Park 1 has a peculiar acoustic environment since it always has background music in its whole extension. In case of background music could be associated with a better acoustic perception by humans, it could be used as a strategy to improve the comfort level in similar parks.

The multi-domain analysis highlighted an existing dependency among different domains in both parks, but these are more evident in Park 1 than in Park 2, which could be due to the differences between park designs. For example, background music in Park 1 can improve people’s well-being and make them more tolerant of thermal conditions. In detail, noise perception and acoustic quality votes were related to thermal comfort and acceptability in Park 1, while they were not related in Park 2. Alternatively, the better thermal conditions in Park 1 can make people feel better in terms of acoustic and visual. In fact, D’Alessandro et al. (2018) and Lau and Choi (2021) already demonstrated that thermal and acoustic domains are closely related, and Lam et al. (2020) showed a dependence between visual and thermal perceptions, as found in this study in Park 1. Therefore, this research confirms these results from previous studies, which means that environmental aspects can be used as comfort triggers for the other domains that are not directly related to these factors, as suggested by Lam et al. (2020) and Lau and Choi (2021). Park 1, for example, has some elements that can be considered specific triggers for comfort, such as the fountain (to improve visual and acoustic comfort) and the background music (acoustic), but they can also affect the other domains and the overall comfort level. From this, it is evident that a multi-domain analysis is needed when evaluating outdoor human comfort, and it is even more urgent in places with complex designs hosting a variety of functions.

Then, knowing that human comfort assessment should involve all physical stimuli, could this analysis be done only considering physical parameters? Despite the equipment costs, environmental data are usually easier to get and analyze because they do not depend on people. They are useful for understanding human comfort because all physical parameters affect comfort perception somehow. For example, people generally preferred a colder environment in Park 2, while in Park 1, the thermal preference votes were mostly for no changes. This distinction can be explained by the mean air temperature, generally higher in Park 2 than in 1, and the higher wind speed in Park 1. However, sometimes only the physical parameters are not enough to explain human responses. In this study, for example, people expressed higher satisfaction in terms of acoustic quality in Park 1 even though the sound pressure level was about 10 dB higher in this place and higher than national requirements for public leisure places. A hypothesis to justify this outcome is the quality of site-specific sound sources in the two parks. A sound can be louder but more pleasant for people, so they are more tolerant to the higher sound pressure.

Moreover, differences among survey answers between parks were significant in only 14% of cases, even if statistically significant differences were identified in physical parameters. The logistic regression models also clarify that only environmental data are not enough to predict the overall comfort level. Environmental data are important to characterize the places, but human information and feedback are needed to perform a complete evaluation.

Hence, a comprehensive human comfort analysis in outdoor environments should be multi-disciplinary, that is, it should merge environmental (measurement of physical parameters) and social (like surveys or questionnaires) disciplines, and multi-domain, exploring thermal, visual, acoustic, and air quality aspects and the relations among them.

## Conclusions and further developments

Given the lack of comfort experiments addressing the interaction between different comfort domains outdoors, a multi-domain and multi-disciplinary approach to evaluate human comfort in outdoor environments is proposed in this paper. The study aimed at identifying relations between comfort domains and confirmed the need to approach comfort studies from a multi-disciplinary perspective. The method was applied in two urban parks because these spaces are usually related to more comfortable conditions than the urban surrounding. The parks have different designs: Park 1 is more recent and counts with a more innovative design, while Park 2 is a traditional urban green area. Statistical techniques were used to compare them regarding the environmental data and the subjective comfort assessment. No specific discomfort trigger was identified in any park, and people’s overall comfort perception was slightly better in Park 1. In general, the physical parameters differed in the two parks on the same walk, while the survey’s responses were similar in the two places. This demonstrates that human perception of an environment can be similar even under different environmental conditions, emphasizing the importance of including the human dimension in comfort studies by combining environmental and qualitative data.

Dependence among different comfort domains was identified in the two parks, but they are more evident in Park 1 than in Park 2. A hypothesis is that the different park designs can explain this variation. This result confirms the importance of investigating more than one comfort domain together, especially in more modern spaces. The logistic regression models that used qualitative information (survey responses) showed to better predict the overall comfort level than those that used only environmental data. Therefore, a comprehensive human comfort analysis in outdoor environments should comprise not only quantitative but also qualitative techniques under a multi-disciplinary framework, simultaneously investigating the main domains affecting pedestrians’ comfort.

Designing more comfortable outdoor environments is a current challenge for urban planners and finding the crossed effects between comfort domains is essential for facing this issue because it could allow triggering the same comfort perceptions by changing varied environmental factors. Therefore, multi-domain comfort approaches are fundamental for proposing solutions that could enhance urban environmental quality. However, additional monitoring campaigns need to be performed in other seasons besides Summer for providing a comprehensive effectiveness assessment of implemented solutions in the outdoors for human comfort. In fact, human perception can vary under different environmental conditions such as the contribution of natural elements to the energy balance or soundscape. Furthermore, it was already indicated that consecutive exposure to different urban settings influences human perception (Vasilikou and Nikolopoulou 2020). Therefore, the effect of successive diverse exposures during comfort walk experiments on how people perceive and evaluate an environment should be further explored, considering that during a walk people go through varied urban morphologies that affect the environmental conditions and, consequently, their sensation (Lau et al. 2019).

Future developments should also focus on carrying out this multi-domain and multi-disciplinary comfort analysis in outdoor environments other than parks, such as places dedicated to shopping and services. In addition, future studies should further investigate acoustic and visual domains by also considering a sound spectrum analysis and glare incidences, respectively. Visual discomfort sources, perception of sounds from different sources, and questions about perceived air quality could be additionally assessed through the design of dedicated questionnaires.

## Supplementary Information

Below is the link to the electronic supplementary material.

Supplementary file1 (PDF 1.97 MB)
