# Supplementary material for: Multi-domain human-oriented approach to evaluate human comfort in outdoor environments
Source: Int J Biometeorol. 2022 Aug 9;66(10):2033–45. doi: 10.1007/s00484-022-02338-7 (PMC9361257; doi:10.1007/s00484-022-02338-7)
Supplement: Supplementary file 1 — Supplementary file1 (PDF 1.97 MB) [file 484_2022_2338_MOESM1_ESM.pdf]

Multi-domain human-oriented approach to evaluate human comfort in outdoor environments

Cureau, Roberta Jacoby[1]; Pigliautile, Ilaria[1,2]; Kousis, Ioannis[1,2]; Pisello, Anna Laura[1,2]*

[1] CIRIAF – Interuniversity Research Center on Pollution and Environment Mauro Felli – University of Perugia, 06125, Perugia, Italy
[2] Department of Engineering, University of Perugia, Via G. Duranti 93, 06125, Perugia, Italy

* Corresponding author: anna.pisello@unipg.it.

Supplementary Material

This Supplementary Material includes:

- Appendix A: Comfort survey
- Appendix B: Supplemental Figures
- Appendix C: Supplemental Tables
- Appendix D: Logistic regression models and their outcomes

Appendix A. Comfort survey

Gender: □ Male □ Female
Age:
Height:* Weight:*

Mark all appropriate options to indicate your current attire (upper and lower body and footwear):*

□ Upper body:	Lower body:	Footwear:
□ T-shirt	□ Shorts	□ Shoes
□ Short-sleeved shirt	□ Gymnastic pants	□ Boots
□ Long-sleeved shirt	□ Jeans pants	□ Socks
□ Sweater	□ Other long pants	□ Sandals
□ Jacket	□ Short skirt	□ Slippers
□ Dress	□ Long skirt	□ Other (specify):
□ Sunglasses	□ Other (specify):	
□ Hat		
□ Other (specify):		

1 - What is your thermal sensation at this moment in this current spot?
□ Very cold □ Cold □ Neutral □ Hot □ Very hot

2 - How do you feel about the wind at this moment in this current spot?
□ No wind □ Light wind □ Windy □ Very windy □ Too windy

3 - How do you feel about the humidity at this moment in this current spot?
□ Very dry □ Dry □ Neutral □ Humid □ Very humid

4 - How do you feel at this moment in this thermal environment?

□ Very uncomfortable □ Uncomfortable □ Neither uncomfortable nor comfortable □ Comfortable □ Very comfortable

5 - You would feel more comfortable if this environment was:
□ Much colder □ Colder □ No changes □ Hotter □ Much hotter

6 - You would feel more comfortable if this environment was:
□ Much less windy □ Less windy □ No changes □ Windier □ Much windier

7 - You would feel more comfortable if this environment was:
□ Much less humid □ Less humid □ No changes □ More humid □ Much more humid

8 - For you, this thermal environment right now is:
□ Clearly unacceptable □ Just unacceptable □ Neither unacceptable nor acceptable □ Just acceptable □ Clearly acceptable

9 - What is your visual sensation at this moment in this current spot?
□ Very dark □ Dark □ Neutral □ Bright □ Very bright

10 - How do you feel at this moment in this visual environment?
□ Very uncomfortable □ Uncomfortable □ Neither uncomfortable nor comfortable □ Comfortable □ Very comfortable

11 - You would feel more comfortable if this environment was:
□ Much darker □ Darker □ No changes □ Brighter □ Much brighter

12 - For you, this visual environment right now is:
□ Clearly unacceptable □ Just unacceptable □ Neither unacceptable nor acceptable □ Just acceptable □ Clearly acceptable

13 - How do you judge the acoustic environment in which you are?
□ Very silent □ Silent □ Neutral □ Noisy □ Very noisy

14 - How do you describe the current environment sound?
□ Very bad □ Bad □ Neither bad nor good □ Good □ Very good

15 - To what extent is the present surrounding sound environment appropriate to the present place?
□ Not at all □ Slightly □ Moderately □ Very □ Perfectly

16 - At this moment, how do you feel overall in this current spot?
□ Very uncomfortable □ Uncomfortable □ Neither uncomfortable nor comfortable □ Comfortable □ Very comfortable

17 - At this moment, you are:
□ Under shade □ Exposed to sunlight

* Questions that were only included in the participants' survey.

Appendix B. Supplemental Figures

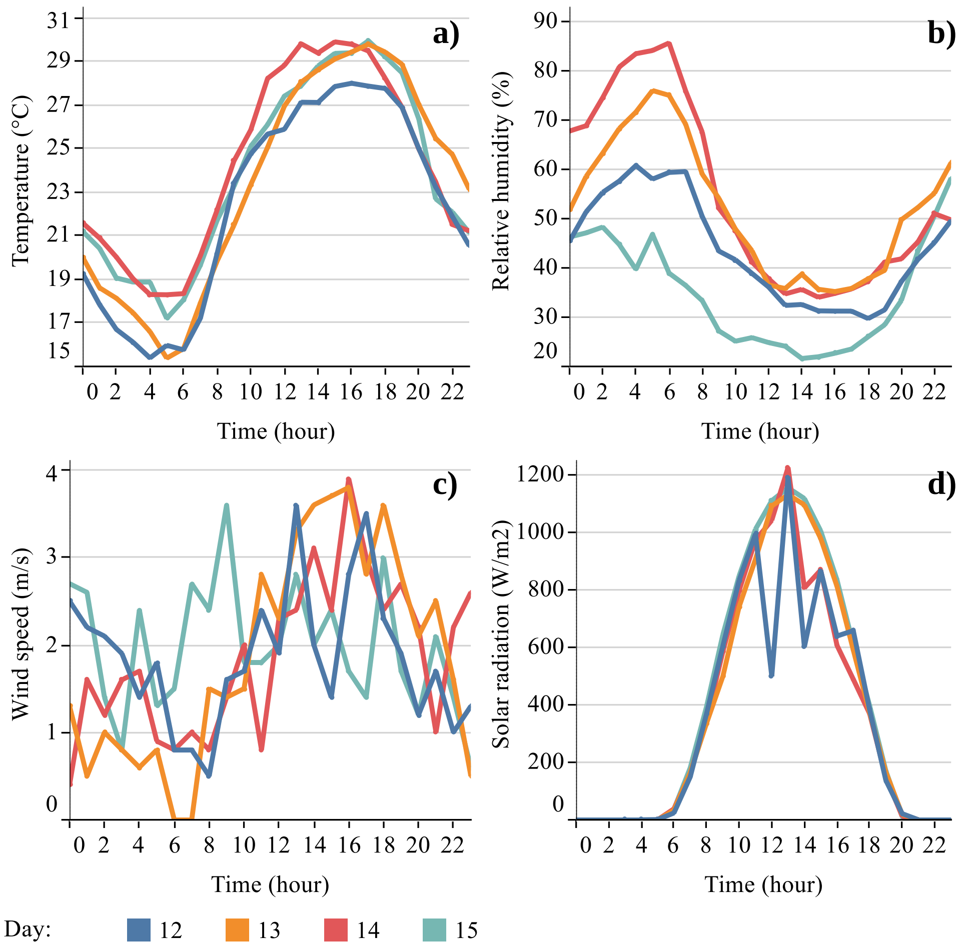

Fig. B1 Parameters measured with the fixed weather station during the four days of experiments: a) temperature; b) relative humidity; c) wind speed; d) solar radiation

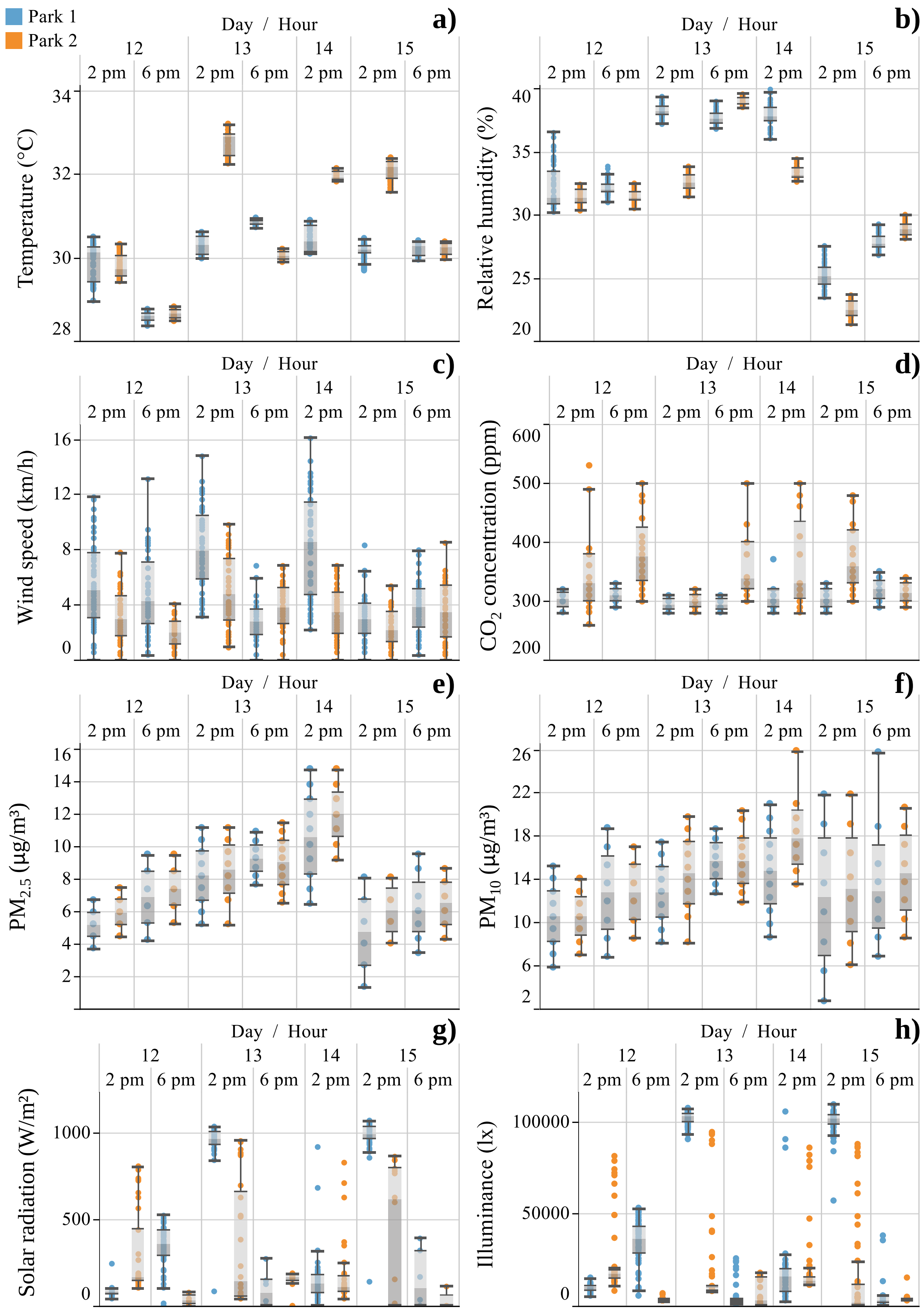

Fig. B2 Environmental data: a) temperature; b) relative humidity; c) wind speed; d) CO_2 concentration; e) $PM_{2.5}$ concentration; f) PM_{10} concentration; g) solar radiation; h) illuminance

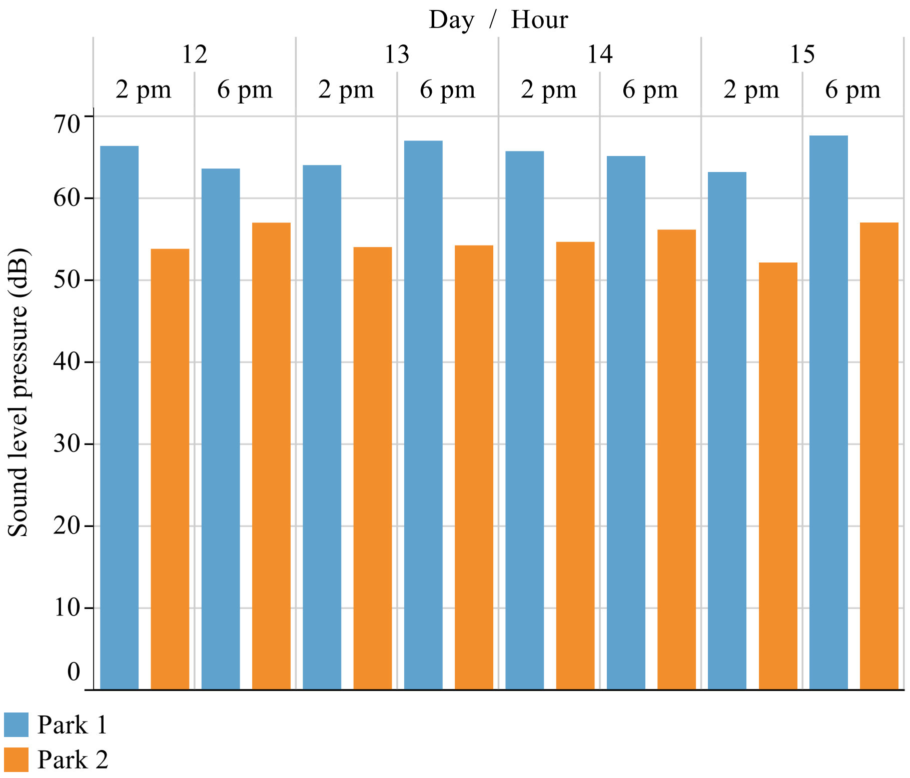

Fig. B3 Sound pressure levels measured in Parks 1 and 2 during each walk

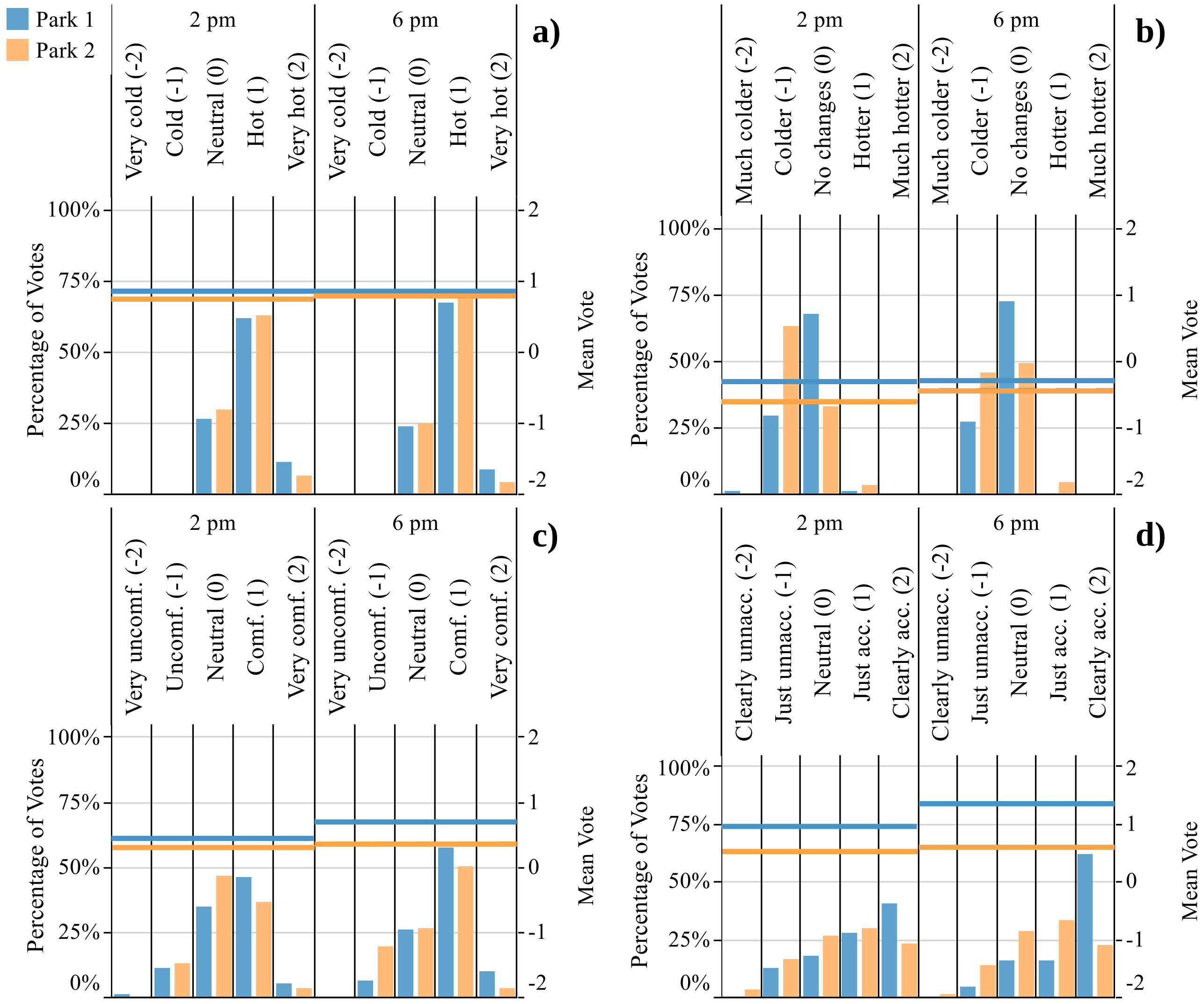

Note: uncomf. means uncomfortable; comf. means comfortable; unnacc. means unacceptable; acc. means acceptable.

Fig. B4 Survey answers related to the thermal domain: a) thermal sensation; b) thermal preference; c) thermal comfort; d) thermal acceptability

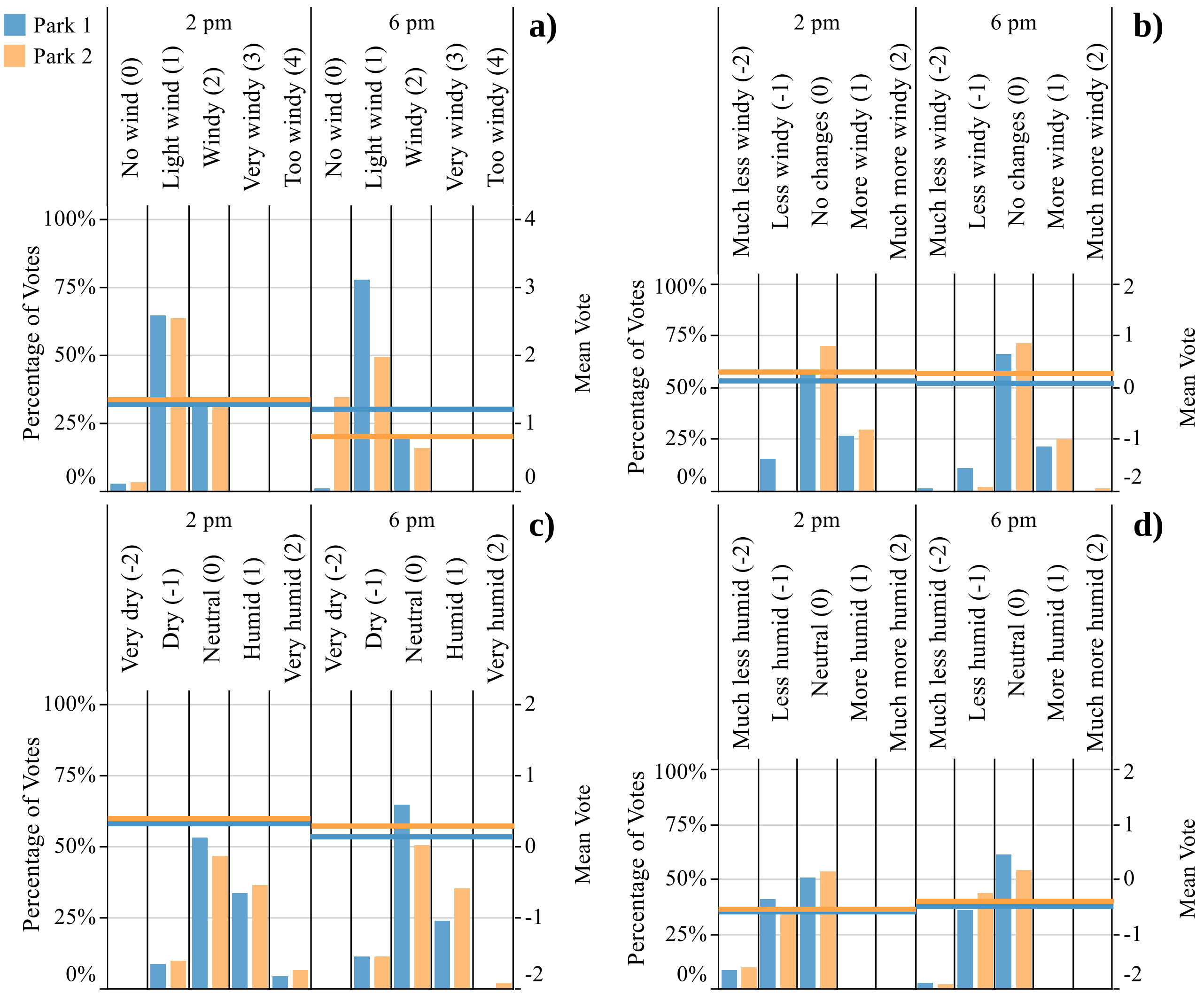

Fig. B5 Survey answers related to the thermal domain for questions regarding wind and humidity: a) wind sensation; b) wind preference; c) humidity sensation; d) humidity preference

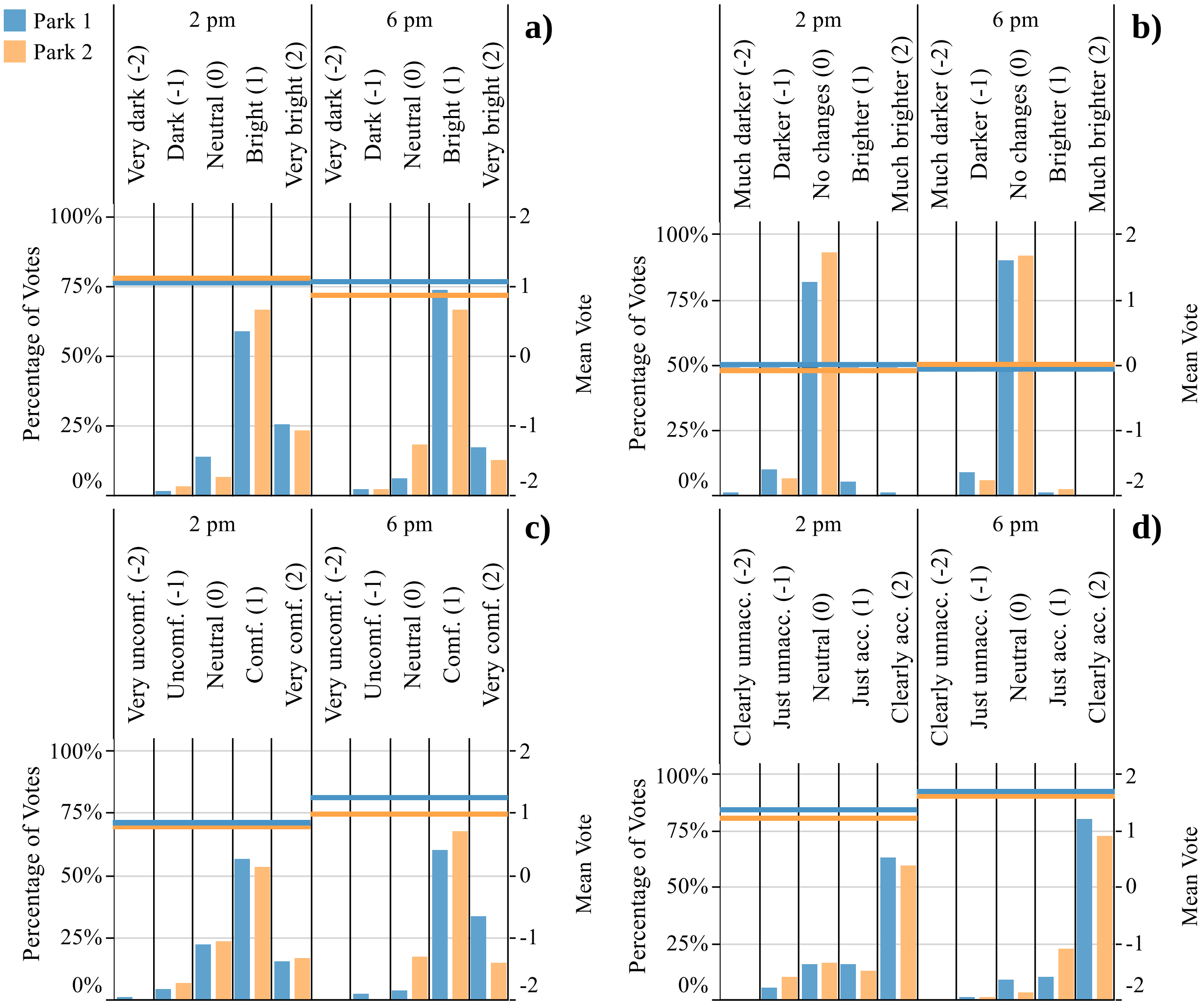

Note: uncomf. means uncomfortable; comf. means comfortable; unnacc. means unacceptable; acc. means acceptable.

Fig. B6 Survey answers related to the visual domain: a) visual sensation; b) visual preference; c) visual comfort; d) visual acceptability

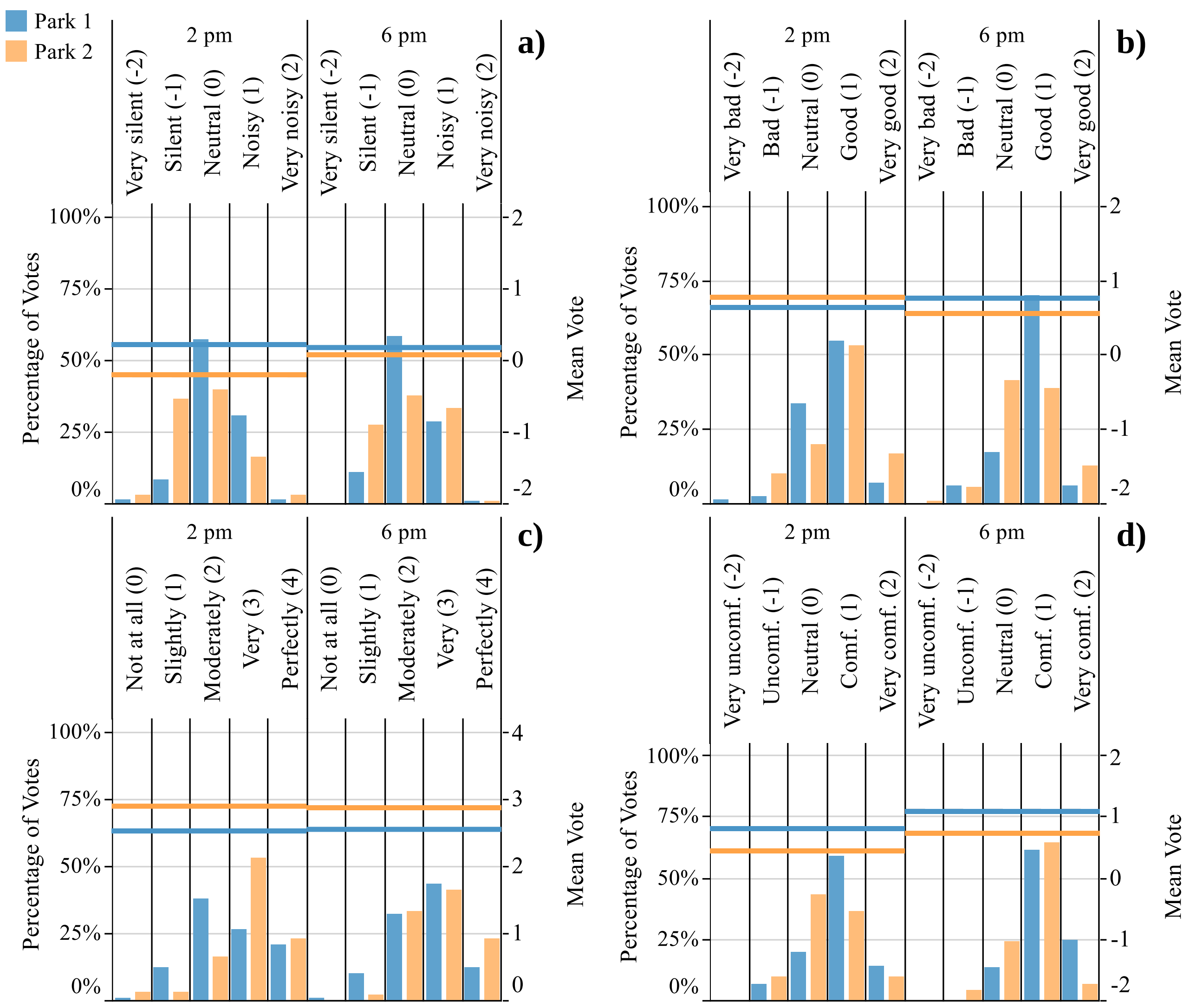

Note: uncomf. means uncomfortable; comf. means comfortable.

Fig. B7 Survey answers related to the acoustic domain and overall comfort: a) noise perception; b) acoustic quality perception; c) whether the acoustic environment is appropriate to the place; d) overall comfort

Appendix C. Supplemental Tables

Table C1 Differences in temperature and relative humidity identified with the fixed weather station and the wearable equipment during each walk

Walk	Temperature (°C)		Relative humidity (%)	
	Station	Wearable	Station	Wearable
June 12th 2 pm	0.37	-0.15	-0.40	-0.70
June 12th 6 pm	-1.01	0.07	3.12	-0.62
June 13th 2 pm	0.75	2.55	-4.82	-5.59
June 13th 6 pm	-0.92	-0.82	5.05	1.33
June 14th 2 pm	0.36	1.50	-1.76	-4.51
June 15th 2 pm	0.68	1.94	0.42	-2.73
June 15th 6 pm	-1.18	0.03	3.68	1.01

Table C2 *p* values for Fisher's exact test

	Thermal sensation	Thermal comfort	Thermal preference	Thermal acceptability	Noise perception	Acoustic quality	Acoustic appropriate
**Park 1:**							
Visual sensation	0.773	0.135	0.021*	0.022*	0.761	0.442	0.732
Visual comfort	0.008*	<0.001*	0.315	0.001*	0.072	0.045*	0.007*
Visual preference	0.194	0.198	0.004*	0.004*	0.891	0.122	0.091
Visual acceptability	0.056	0.003*	0.010*	<0.001*	0.265	0.016*	0.004*
Noise perception	0.260	0.025*	0.225	0.022*	-	-	-
Acoustic quality	0.120	0.011*	0.078	0.001*	-	-	-
Acoustic appropriate	0.026*	0.208	0.732	0.194	-	-	-
**Park 2:**							
Visual sensation	0.211	0.020*	0.115	0.192	0.116	0.425	0.247
Visual comfort	0.536	0.379	0.418	0.010*	0.860	0.013*	0.291
Visual preference	0.256	0.367	0.198	0.104	0.873	0.401	0.528
Visual acceptability	0.663	0.203	0.227	<0.001*	0.134	0.039*	0.021*
Noise perception	0.620	0.218	0.942	0.681	-	-	-
Acoustic quality	0.389	0.537	0.742	0.187	-	-	-
Acoustic appropriate	0.092	0.274	0.017*	0.065	-	-	-

Note: * $p < 0.05$.

Appendix D. Logistic regression models and their outcomes

This Appendix presents the details related to the logistic regression models. The aim of this analysis was to verify which kind of independent variable would be better to predict the overall comfort vote (dependent variable). Six models were developed, and the independent variables used in each of them are presented in Table D1.

Table D1 Independent variables included in each multinomial logistic regression model

**Model**	**Independent variables**	
Model 1	Demographic information	Age and gender
Model 2	Monitored environmental data	Temperature, relative humidity, wind speed, solar radiation, illuminance, CO_2 concentration, $PM_{2.5}$, PM_{10}, and sound pressure level
Model 3	Questions related to the thermal domain	Thermal sensation, comfort, preference, and acceptability
Model 4	Questions related to the visual domain	Visual sensation, comfort, preference, and acceptability
Model 5	Questions related to the acoustic domain	Noise and acoustic quality perception, whether the sound environment is appropriate to the place
Model 6	Questions related to thermal, visual, and acoustic domains	Thermal sensation, comfort, preference, and acceptability; visual sensation, comfort, preference, and acceptability; noise and acoustic quality perception, whether the sound environment is appropriate to the place

The environmental data are the parameter means in each park during the respective walk. As these data are numeric, they do not require a reference level for the model. All the other independent variables are categorical and come from participants' and local people's responses to the survey. The reference level for gender was "female". People's ages were divided into seven levels (equal or less to 20, 21-30, 31-40, 41-50, 51-60, 61-70, and 71-80), being the level "equal or less than 20 years old" the reference. Following their respective answer options, questions were divided into five levels, and the central level (i.e., neutral) was adopted as the reference for all the models. Models from the same park were evaluated and compared using the McFadden pseudo R^2. The following tables present the outcomes for each logistic regression model.

Table D2 Model 1 in Park 1 (demographic information)

Variable		Overall comfort level	Coefficient	Standard error	Confidence intervals 2.5%	97.5%	p value
(Intercept)		-1	-19.187	0.315	-19.804	-18.570	
		1	1.276	0.527	0.244	2.309	
		2	-0.177	0.672	-1.494	1.140	
Age:	< 20	Reference					0.245
	21-30	-1	18.892	0.315	18.275	19.509	
		1	-0.506	0.618	-1.717	0.705	
		2	-0.400	0.793	-1.956	1.155	
	31-40	-1	-6.186	0.000	-6.186	-6.186	
		1	-0.286	0.836	-1.924	1.352	
		2	-0.349	1.115	-2.534	1.836	
	41-50	-1	-1.851	0.000	-1.851	-1.851	
		1	0.671	0.902	-1.097	2.439	
		2	1.282	1.024	-0.724	3.289	
	51-60	-1	-4.342	0.000	-4.342	-4.342	
		1	-0.351	0.765	-1.850	1.147	
		2	0.389	0.906	-1.387	2.165	
	61-70	-1	0.048	0.000	0.048	0.048	
		1	12.727	0.661	11.431	14.024	
		2	14.860	0.661	13.564	16.156	
	71-80	-1	0.770	0.000	0.770	0.770	
		1	12.593	0.000	12.593	12.593	
		2	-6.471	0.000	-6.471	-6.471	
Gender:	Female	Reference					0.287
	Male	-1	-1.433	1.209	-3.803	0.937	
		1	0.414	0.476	-0.520	1.347	
		2	0.456	0.576	-0.672	1.584	
AIC		336.5					
McFadden pseudo R^2		0.074					

Table D3 Model 2 in Park 1 (monitored environmental data)

Variable	Overall comfort level	Coefficient	Standard error	Confidence intervals		*p* value
				2.5%	97.5%	
(Intercept)	-1	0.039	0.000	0.039	0.039	1
	1	-0.001	0.000	-0.001	-0.001	
	2	-0.004	0.000	-0.004	-0.004	
Temperature	-1	10.272	0.000	10.271	10.272	1
	1	-0.465	0.000	-0.465	-0.464	
	2	-4.435	0.000	-4.435	-4.434	
Relative humidity	-1	-4.215	0.000	-4.216	-4.215	1
	1	0.287	0.001	0.285	0.289	
	2	2.517	0.001	2.515	2.519	
Wind speed	-1	5.485	0.000	5.485	5.485	1
	1	-0.236	0.000	-0.236	-0.235	
	2	-3.999	0.000	-3.999	-3.998	
Solar radiation	-1	0.350	0.000	0.350	0.351	1
	1	0.011	0.006	-0.001	0.023	
	2	-0.119	0.006	-0.130	-0.108	
Illuminance	-1	-0.004	0.000	-0.004	-0.004	1
	1	0.000	0.000	0.000	0.000	
	2	0.001	0.000	0.001	0.001	
CO_2 concentration	-1	-1.153	0.002	-1.156	-1.149	1
	1	0.044	0.001	0.042	0.047	
	2	0.265	0.001	0.262	0.267	
PM2.5	-1	7.838	0.000	7.838	7.838	1
	1	-0.402	0.000	-0.403	-0.401	
	2	-5.456	0.000	-5.457	-5.456	
PM10	-1	-5.646	0.000	-5.646	-5.646	1
	1	0.184	0.000	0.183	0.185	
	2	1.048	0.000	1.047	1.048	
Sound pressure level	-1	2.584	0.000	2.583	2.584	1
	1	-0.088	0.000	-0.089	-0.088	
	2	0.095	0.000	0.094	0.096	
AIC	281.2					
McFadden pseudo R^2	0.126					

Table D4 Model 3 in Park 1 (questions related to thermal domain)

Variable		Overall comfort level	Coefficient	Standard error	Confidence intervals		*p* value
					2.5%	97.5%	
(Intercept)		-1	-0.253	1.861	-3.902	3.395	
		1	1.257	0.918	-0.542	3.055	
		2	-0.863	1.282	-3.376	1.649	
Thermal sensation:	0	Reference					0.488
	1	-1	-2.084	1.676	-5.369	1.200	
		1	0.714	0.732	-0.720	2.149	
		2	-0.155	0.838	-1.797	1.487	
	2	-1	-2.040	2.085	-6.126	2.046	
		1	-0.029	0.997	-1.983	1.924	
		2	-0.527	1.371	-3.213	2.160	
Thermal comfort:	0	Reference					0.001
	-2	-1	29.545	0.000	29.545	29.545	
		1	-9.453	0.000	-9.453	-9.453	
		2	-1.971	0.000	-1.971	-1.971	
	-1	-1	1.300	1.462	-1.565	4.165	
		1	-0.979	0.863	-2.670	0.712	
		2	0.064	1.362	-2.605	2.734	
	1	-1	-14.468	0.000	-14.468	-14.468	
		1	0.747	0.706	-0.636	2.130	
		2	0.973	0.971	-0.931	2.877	
	2	-1	1.316	0.000	1.316	1.316	
		1	17.792	0.661	16.496	19.089	
		2	21.695	0.661	20.399	22.992	
Thermal preference:	0	Reference					0.193
	-2	-1	-21.335	0.000	-21.335	-21.335	
		1	-27.857	0.000	-27.857	-27.857	
		2	-15.261	0.000	-15.261	-15.261	
	-1	-1	-0.430	1.519	-3.407	2.547	
		1	-1.782	0.611	-2.979	-0.585	
		2	-0.667	0.817	-2.269	0.936	
	1	-1	-1.587	0.000	-1.587	-1.587	
		1	12.624	0.000	12.624	12.624	
		2	-1.670	0.000	-1.670	-1.670	
Thermal acceptability:	0	Reference					0.698
	-1	-1	0.094	1.582	-3.006	3.194	
		1	-0.275	0.872	-1.984	1.434	
		2	-0.201	1.383	-2.911	2.510	
	1	-1	0.921	1.454	-1.928	3.770	
		1	0.427	0.731	-1.006	1.861	
		2	-0.468	1.372	-3.157	2.220	
	2	-1	-13.494	0.000	-13.494	-13.494	
		1	0.034	0.828	-1.588	1.657	
		2	1.195	1.152	-1.062	3.452	
AIC		298.2					
McFadden pseudo R^2		0.297					

Table D5 Model 4 in Park 1 (questions related to visual domain)

Variable		Overall comfort level	Coefficient	Standard error	Confidence intervals 2.5%	97.5%	p value
(Intercept)		-1	-89.232	0.934	-91.062	-87.402	
		1	-1.643	0.988	-3.579	0.293	
		2	-90.338	1.124	-92.542	-88.134	
Visual sensation:	0	Reference					0.478
	-1	-1	-34.631	0.000	-34.631	-34.631	
		1	-1.259	1.731	-4.651	2.133	
		2	-69.729	0.000	-69.729	-69.729	
	1	-1	28.266	0.462	27.360	29.172	
		1	1.368	0.825	-0.250	2.986	
		2	0.986	1.291	-1.544	3.516	
	2	-1	71.919	0.000	71.919	71.919	
		1	1.027	1.022	-0.977	3.030	
		2	0.439	1.503	-2.506	3.384	
Visual comfort:	0	Reference					0.002
	-2	-1	73.351	NaN	NaN	NaN	
		1	-41.813	NaN	NaN	NaN	
		2	0.476	0.000	0.476	0.476	
	-1	-1	-87.710	0.000	-87.710	-87.710	
		1	-2.008	1.755	-5.449	1.432	
		2	-19.326	0.000	-19.326	-19.326	
	1	-1	-63.072	0.462	-63.978	-62.166	
		1	1.396	0.744	-0.062	2.854	
		2	1.309	1.252	-1.144	3.763	
	2	-1	-126.041	0.000	-126.041	-126.041	
		1	1.316	1.018	-0.678	3.311	
		2	3.008	1.435	0.196	5.821	
Visual preference:	0	Reference					0.563
	-2	-1	-23.490	0.000	-23.490	-23.490	
		1	-67.429	0.000	-67.429	-67.429	
		2	-9.688	0.000	-9.688	-9.688	
	-1	-1	3.111	2.332	-1.460	7.683	
		1	1.842	1.361	-0.826	4.509	
		2	-42.269	0.000	-42.269	-42.269	
	1	-1	-43.632	0.000	-43.632	-43.632	
		1	-0.435	1.341	-3.063	2.192	
		2	-38.771	0.000	-38.771	-38.771	
	2	-1	-71.447	0.000	-71.447	-71.447	
		1	-126.680	0.000	-126.680	-126.680	
		2	-74.036	0.000	-74.036	-74.036	
Visual acceptability:	0	Reference					0.006
	-1	-1	88.681	1.165	86.398	90.965	
		1	1.111	1.688	-2.199	4.420	
		2	-4.039	0.000	-4.039	-4.039	
	1	-1	124.022	0.462	123.116	124.928	
		1	1.128	1.058	-0.945	3.202	
		2	87.546	1.007	85.573	89.520	
	2	-1	28.890	0.000	28.890	28.890	
		1	1.109	0.756	-0.372	2.591	
		2	88.788	0.726	87.365	90.212	
AIC		305.0					
McFadden pseudo R^2		0.313					

Table D6 Model 5 in Park 1 (questions related to acoustic domain)

Variable		Overall comfort level	Coefficient	Standard error	Confidence intervals 2.5%	97.5%	p value
(Intercept)		-1	-1.311	0.925	-3.123	0.502	
		1	0.681	0.497	-0.293	1.655	
		2	-25.289	0.741	-26.741	-23.836	
Noise perception:	0	Reference					0.902
	-2	-1	0.549	NaN	NaN	NaN	
		1	14.038	0.000	14.038	14.038	
		2	-10.193	0.000	-10.193	-10.193	
	-1	-1	-15.241	0.000	-15.241	-15.241	
		1	-0.610	1.243	-3.046	1.826	
		2	0.043	1.334	-2.572	2.659	
	1	-1	0.241	1.126	-1.965	2.447	
		1	-0.315	0.606	-1.503	0.874	
		2	0.036	0.850	-1.630	1.701	
	2	-1	31.828	506.393	-960.685	1024.341	
		1	17.466	507.957	-978.111	1013.043	
		2	-1.131	0.000	-1.131	-1.131	
Acoustic quality perception:	0	Reference					<0.001
	-2	-1	-16.976	0.000	-16.976	-16.976	
		1	-14.562	0.000	-14.562	-14.562	
		2	-1.036	0.000	-1.036	-1.036	
	-1	-1	-29.163	12.243	-53.159	-5.166	
		1	0.788	1.088	-1.345	2.920	
		2	-5.850	0.000	-5.850	-5.850	
	1	-1	0.705	1.340	-1.921	3.331	
		1	2.642	0.678	1.313	3.971	
		2	25.176	0.609	23.983	26.370	
	2	-1	-0.081	0.000	-0.081	-0.081	
		1	25.182	0.445	24.311	26.054	
		2	49.480	0.445	48.608	50.351	
Sound environment appropriate:	2	Reference					0.006
	0	-1	-18.822	0.000	-18.822	-18.822	
		1	-37.971	0.000	-37.971	-37.971	
		2	0.113	1.908	-3.628	3.853	
	1	-1	-0.293	1.329	-2.897	2.311	
		1	-1.793	0.863	-3.484	-0.102	
		2	-0.238	1.671	-3.512	3.036	
	3	-1	-24.607	0.000	-24.607	-24.607	
		1	-0.616	0.681	-1.951	0.719	
		2	1.845	1.263	-0.630	4.320	
	4	-1	-17.869	0.000	-17.869	-17.869	
		1	-0.377	1.252	-2.830	2.076	
		2	2.714	1.635	-0.492	5.919	
AIC	305.0						
McFadden pseudo R^2	0.328						

Table D7 Model 6 in Park 1 (questions related to thermal, visual, and acoustic domain)

Variable		Overall comfort level	Coefficient	Standard error	Confidence intervals 2.5%	97.5%	*p* value
(Intercept)		-1	-377.943	0.000	-377.943	-377.943	
		1	-2493.431	1.118	-2495.622	-2491.240	
		2	-5495.874	1.118	-5498.065	-5493.683	
Thermal sensation:	0	Reference					<0.001
	1	-1	-1174.788	0.000	-1174.788	-1174.788	
		1	2600.943	0.426	2600.109	2601.777	
		2	2600.232	0.426	2599.398	2601.066	
	2	-1	-1344.890	NaN	NaN	NaN	
		1	2707.924	0.853	2706.251	2709.596	
		2	2706.898	0.853	2705.225	2708.570	
Thermal comfort:	0	Reference					<0.001
	-2	-1	2153.535	0.000	2153.535	2153.535	
		1	-332.263	0.000	-332.263	-332.263	
		2	39.730	0.000	39.730	39.730	
	-1	-1	1896.903	0.000	1896.903	1896.903	
		1	998.516	1.741	995.103	1001.929	
		2	1001.302	1.741	997.889	1004.715	
	1	-1	-1293.704	NaN	NaN	NaN	
		1	1530.327	0.646	1529.060	1531.593	
		2	1532.479	0.646	1531.212	1533.745	
	2	-1	475.508	0.000	475.508	475.508	
		1	2307.945	0.993	2305.998	2309.892	
		2	2313.719	0.993	2311.772	2315.665	
Thermal preference:	0	Reference					<0.001
	-2	-1	-625.010	0.000	-625.010	-625.010	
		1	-3053.289	0.000	-3053.289	-3053.289	
		2	111.909	0.000	111.909	111.909	
	-1	-1	-1505.822	0.000	-1505.822	-1505.822	
		1	-2441.563	0.665	-2442.866	-2440.261	
		2	-2439.455	0.665	-2440.758	-2438.152	
	1	-1	140.623	NaN	NaN	NaN	
		1	-19.600	0.000	-19.600	-19.600	
		2	-144.401	0.000	-144.401	-144.401	
Thermal acceptability:	0	Reference					<0.001
	-1	-1	755.770	NaN	NaN	NaN	
		1	-856.484	1.642	-859.702	-853.266	
		2	-856.458	1.642	-859.676	-853.241	
	1	-1	-863.885	0.000	-863.885	-863.885	
		1	-1422.307	1.061	-1424.387	-1420.226	
		2	-1422.812	1.061	-1424.893	-1420.732	
	2	-1	-682.709	0.000	-682.709	-682.709	
		1	-2160.123	1.014	-2162.111	-2158.134	
		2	-2159.674	1.014	-2161.663	-2157.686	
Visual sensation:	0	Reference					0.838
	-1	-1	-476.262	0.000	-476.262	-476.262	
		1	-381.786	0.000	-381.786	-381.786	
		2	-1180.870	0.000	-1180.870	-1180.870	
	1	-1	-108.940	0.000	-108.940	-108.940	
		1	-944.029	1.327	-946.629	-941.429	
		2	-943.807	1.327	-946.406	-941.207	
	2	-1	582.731	NaN	NaN	NaN	
		1	-1037.217	1.406	-1039.971	-1034.462	
		2	-1036.975	1.406	-1039.729	-1034.220	
Visual comfort:	0	Reference					<0.001

	-2	-1	3161.016	NaN	NaN	NaN	
		1	-469.607	0.000	-469.607	-469.607	
		2	88.063	0.000	88.063	88.063	
	-1	-1	-2527.718	0.000	-2527.718	-2527.718	
		1	-1409.853	0.000	-1409.853	-1409.853	
		2	224.359	0.000	224.359	224.359	
	1	-1	881.327	0.000	881.327	881.327	
		1	2787.996	1.283	2785.481	2790.511	
		2	2788.156	1.283	2785.641	2790.671	
	2	-1	-543.763	NaN	NaN	NaN	
		1	936.955	1.255	934.495	939.415	
		2	938.352	1.255	935.892	940.811	
Visual preference:	0	Reference					0.014
	-2	-1	-517.700	NaN	NaN	NaN	
		1	-2960.976	0.000	-2960.976	-2960.976	
		2	-285.027	0.000	-285.027	-285.027	
	-1	-1	1847.848	0.000	1847.848	1847.848	
		1	2589.724	0.000	2589.724	2589.724	
		2	-1137.905	0.000	-1137.905	-1137.905	
	1	-1	-1478.738	0.000	-1478.738	-1478.738	
		1	209.382	0.000	209.382	209.382	
		2	-311.083	0.000	-311.083	-311.083	
	2	-1	-625.010	0.000	-625.010	-625.010	
		1	-3053.289	0.000	-3053.289	-3053.289	
		2	111.909	0.000	111.909	111.909	
Visual acceptability:	0	Reference					<0.001
	-1	-1	2580.328	NaN	NaN	NaN	
		1	200.705	0.000	200.705	200.705	
		2	403.745	0.000	403.745	403.745	
	1	-1	2899.820	0.000	2899.820	2899.820	
		1	1614.758	1.108	1612.587	1616.929	
		2	2577.223	1.108	2575.052	2579.394	
	2	-1	2355.164	0.000	2355.164	2355.164	
		1	2451.343	0.559	2450.248	2452.438	
		2	3416.867	0.559	3415.772	3417.962	
Noise perception:	0	Reference					0.197
	-2	-1	223.637	0.000	223.637	223.637	
		1	203.036	0.000	203.036	203.036	
		2	-492.441	0.000	-492.441	-492.441	
	-1	-1	-348.776	NaN	NaN	NaN	
		1	-501.660	0.750	-503.130	-500.189	
		2	-500.172	0.750	-501.643	-498.702	
	1	-1	-1420.141	0.000	-1420.141	-1420.141	
		1	-518.814	0.456	-519.708	-517.919	
		2	-519.590	0.456	-520.484	-518.695	
	2	-1	2092.466	0.000	2092.466	2092.466	
		1	2520.303	0.000	2520.303	2520.303	
		2	184.222	0.000	184.222	184.222	
Acoustic quality perception:	0	Reference					<0.001
	-2	-1	-143.343	NaN	NaN	NaN	
		1	-1006.278	0.000	-1006.278	-1006.278	
		2	54.660	0.000	54.660	54.660	
	-1	-1	-844.692	0.000	-844.692	-844.692	
		1	1762.001	0.000	1762.001	1762.001	
		2	-675.034	0.000	-675.034	-675.034	
	1	-1	-426.087	0.000	-426.087	-426.087	
		1	1903.026	0.627	1901.798	1904.255	

		2	3934.268	0.627	3933.040	3935.496	
	2	-1	77.013	0.000	77.013	77.013	
		1	3781.913	0.647	3780.646	3783.180	
		2	5813.886	0.647	5812.618	5815.153	
Sound environment appropriate:	2	Reference					<0.001
	0	-1	-590.801	0.000	-590.801	-590.801	
		1	-3597.945	0.000	-3597.944	-3597.944	
		2	539.399	0.000	539.399	539.399	
	1	-1	-1682.256	0.000	-1682.256	-1682.256	
		1	-2463.383	1.364	-2466.058	-2460.709	
		2	-2464.263	1.364	-2466.937	-2461.589	
	3	-1	-1093.267	0.000	-1093.267	-1093.267	
		1	-683.251	0.717	-684.656	-681.845	
		2	-681.218	0.717	-682.624	-679.813	
	4	-1	1301.274	0.000	1301.274	1301.274	
		1	1450.293	0.756	1448.812	1451.774	
		2	1453.087	0.756	1451.606	1454.568	
AIC	284.5						
McFadden pseudo R^2	0.820						

Table D8 Model 1 in Park 2 (demographic information)

Variable		Overall comfort level	Coefficient	Standard error	Confidence intervals		p value
					2.5%	97.5%	
(Intercept)		-1	-14.443	0.612	-15.641	-13.244	
		1	2.018	1.083	-0.104	4.141	
		2	0.010	1.445	-2.822	2.842	
Age:	< 20	Reference					0.067
	21-30	-1	12.346	0.724	10.928	13.765	
		1	-1.444	1.146	-3.691	0.802	
		2	-33.721	0.000	-33.721	-33.721	
	31-40	-1	12.771	0.645	11.507	14.036	
		1	-2.089	1.166	-4.376	0.197	
		2	-1.697	1.625	-4.882	1.487	
	41-50	-1	12.382	0.888	10.642	14.122	
		1	-1.421	1.188	-3.749	0.908	
		2	-1.097	1.634	-4.299	2.106	
	51-60	-1	-17.422	0.000	-17.422	-17.422	
		1	-1.508	1.251	-3.961	0.945	
		2	-0.689	1.663	-3.948	2.571	
	61-70	-1	-6.148	0.000	-6.148	-6.148	
		1	0.510	1.494	-2.418	3.439	
		2	0.689	1.874	-2.984	4.363	
	71-80	-1	36.137	0.699	34.767	37.507	
		1	21.832	0.699	20.463	23.202	
		2	-7.391	0.000	-7.391	-7.391	
Gender:	Female	Reference					0.851
	Male	-1	0.530	0.940	-1.312	2.372	
		1	-0.209	0.466	-1.122	0.705	
		2	-0.027	0.811	-1.616	1.562	
AIC		261.4					
McFadden pseudo R^2		0.127					

Table D9 Model 2 in Park 2 (monitored environmental data)

Variable	Overall comfort level	Coefficient	Standard error	Confidence intervals 2.5%	97.5%	p value
(Intercept)	-1	-0.004	0.000	0.017	0.018	1
	1	0.003	0.001	-0.002	0.001	
	2	-0.043	0.001	-0.012	-0.008	
Temperature	-1	0.386	0.003	1.047	1.059	1
	1	-0.193	0.031	-0.242	-0.121	
	2	1.654	0.038	1.286	1.428	
Relative humidity	-1	0.686	0.059	0.934	1.151	1
	1	0.178	0.056	0.057	0.266	
	2	-1.318	0.026	-1.217	-1.124	
Wind speed	-1	0.060	0.007	-0.014	0.012	1
	1	-0.145	0.008	-0.082	-0.052	
	2	1.576	0.011	0.691	0.730	
Solar radiation	-1	-0.150	0.036	-0.284	-0.143	1
	1	-0.002	0.015	-0.031	0.027	
	2	0.263	0.028	0.211	0.321	
Illuminance	-1	0.001	0.000	0.001	0.002	1
	1	0.000	0.000	0.000	0.000	
	2	-0.004	0.000	-0.004	-0.003	
CO_2 concentration	-1	0.331	0.011	0.284	0.324	1
	1	-0.004	0.011	-0.026	0.016	
	2	0.087	0.020	0.062	0.140	
PM2.5	-1	0.177	0.018	-0.088	-0.024	1
	1	-0.199	0.022	-0.151	-0.060	
	2	2.400	0.005	1.356	1.372	
PM10	-1	0.859	0.012	-0.349	-0.271	1
	1	-0.030	0.016	-0.134	-0.060	
	2	0.130	0.022	1.011	1.122	
Sound pressure level	-1	-2.905	0.019	-3.061	-2.992	1
	1	0.068	0.052	-0.023	0.184	
	2	-0.809	0.071	-1.031	-0.767	
AIC	229.4					
McFadden pseudo R^2	0.111					

Table D10 Model 3 in Park 2 (questions related to thermal domain)

Variable		Overall comfort level	Coefficient	Standard error	Confidence intervals 2.5%	97.5%	*p* value
(Intercept)		-1	-382.342	0.320	-382.970	-381.714	
		1	-0.623	0.823	-2.235	0.990	
		2	-214.260	0.996	-216.211	-212.308	
Thermal sensation:	0	Reference					0.193
	1	-1	122.708	0.320	122.080	123.336	
		1	0.592	0.896	-1.166	2.349	
		2	1.329	1.247	-1.116	3.773	
	2	-1	442.142	0.000	442.142	442.142	
		1	249.740	0.000	249.740	249.740	
		2	12.651	0.000	12.651	12.651	
Thermal comfort:	0	Reference					0.060
	-1	-1	1.965	1.479	-0.934	4.864	
		1	0.916	0.830	-0.710	2.543	
		2	-10.439	0.000	-10.439	-10.439	
	1	-1	-213.495	NaN	NaN	NaN	
		1	1.967	0.818	0.364	3.571	
		2	1.762	1.428	-1.036	4.561	
	2	-1	-2.438	NaN	NaN	NaN	
		1	235.430	0.843	233.778	237.083	
		2	237.215	0.843	235.563	238.868	
Thermal preference:	0	Reference					0.347
	-1	-1	44.816	0.320	44.188	45.444	
		1	-0.876	0.885	-2.611	0.859	
		2	-2.104	1.460	-4.966	0.757	
	1	-1	109.305	0.000	109.305	109.305	
		1	-1.990	1.633	-5.191	1.211	
		2	-288.139	0.000	-288.139	-288.139	
Thermal acceptability:	0	Reference					0.010
	-2	-1	30.087	NaN	NaN	NaN	
		1	-329.235	0.000	-329.235	-329.235	
		2	11.247	0.000	11.247	11.247	
	-1	-1	212.405	0.860	210.720	214.090	
		1	-2.039	1.190	-4.372	0.294	
		2	59.329	0.000	59.329	59.329	
	1	-1	214.561	0.853	212.889	216.233	
		1	2.014	0.840	0.369	3.660	
		2	213.778	0.779	212.250	215.305	
	2	-1	111.657	0.000	111.657	111.657	
		1	1.490	1.045	-0.558	3.539	
		2	213.372	0.876	211.656	215.088	
AIC		207.6					
McFadden pseudo R^2		0.445					

Table D11 Model 4 in Park 2 (questions related to visual domain)

Variable		Overall comfort level	Coefficient	Standard error	Confidence intervals 2.5%	97.5%	*p* value
(Intercept)		-1	1.952	1.763	-1.503	5.406	
		1	1.226	1.218	-1.162	3.613	
		2	-66.114	0.317	-66.736	-65.492	
Visual sensation:	0	Reference					0.075
	-1	-1	31.183	0.864	29.489	32.877	
		1	29.009	0.864	27.315	30.703	
		2	-0.999	0.000	-0.999	-0.999	
	1	-1	-1.534	1.301	-4.084	1.017	
		1	-0.355	0.667	-1.661	0.952	
		2	28.247	0.610	27.051	29.443	
	2	-1	-4.399	2.364	-9.031	0.234	
		1	-1.727	0.887	-3.466	0.012	
		2	26.406	0.731	24.974	27.838	
Visual comfort:	0	Reference					0.209
	-1	-1	-34.277	0.000	-34.277	-34.277	
		1	-9.116	0.000	-9.116	-9.116	
		2	-4.428	NaN	NaN	NaN	
	1	-1	0.102	1.253	-2.354	2.558	
		1	0.748	0.597	-0.423	1.919	
		2	25.146	0.540	24.087	26.204	
	2	-1	-20.710	0.000	-20.710	-20.710	
		1	2.192	1.016	0.201	4.182	
		2	27.591	0.639	26.339	28.843	
Visual preference:	0	Reference					0.596
	-1	-1	1.895	1.631	-1.302	5.091	
		1	-0.272	1.128	-2.483	1.940	
		2	-27.014	0.000	-27.014	-27.014	
	1	-1	-12.165	0.000	-12.165	-12.165	
		1	30.374	0.000	30.374	30.374	
		2	-1.724	0.000	-1.724	-1.724	
Visual acceptability:	0	Reference					0.585
	-1	-1	-1.417	2.301	-5.926	3.092	
		1	-40.549	0.000	-40.549	-40.549	
		2	-19.379	NaN	NaN	NaN	
	1	-1	-2.732	1.813	-6.285	0.821	
		1	-0.766	1.163	-3.045	1.513	
		2	12.148	0.738	10.701	13.594	
	2	-1	-3.266	1.763	-6.722	0.191	
		1	-0.724	1.192	-3.061	1.612	
		2	11.707	0.564	10.601	12.813	
AIC		268.0					
McFadden pseudo R^2		0.198					

Table D12 Model 5 in Park 2 (questions related to acoustic domain)

Variable		Overall comfort level	Coefficient	Standard error	Confidence intervals		*p* value
					2.5%	97.5%	
(Intercept)		-1	-0.424	0.996	-2.376	1.528	
		1	1.382	0.603	0.200	2.564	
		2	-64.929	0.489	-65.888	-63.971	
Noise perception:	0	Reference					0.550
	-2	-1	-0.643	NaN	NaN	NaN	
		1	39.598	NaN	NaN	NaN	
		2	-1.772	0.000	-1.772	-1.772	
	-1	-1	0.817	1.168	-1.472	3.106	
		1	-1.289	0.636	-2.536	-0.042	
		2	0.086	1.077	-2.025	2.197	
	1	-1	-1.316	1.364	-3.989	1.357	
		1	-0.668	0.578	-1.801	0.464	
		2	-0.008	1.444	-2.838	2.822	
	2	-1	-2.898	0.000	-2.898	-2.898	
		1	19.209	0.000	19.209	19.209	
		2	0.665	0.000	0.665	0.665	
Acoustic quality perception:	0	Reference					0.120
	-2	-1	-3.133	0.000	-3.133	-3.133	
		1	14.803	0.000	14.803	14.803	
		2	0.249	0.000	0.249	0.249	
	-1	-1	-53.241	0.000	-53.241	-53.241	
		1	-2.335	1.281	-4.846	0.176	
		2	-1.868	0.000	-1.868	-1.868	
	1	-1	0.140	1.181	-2.174	2.454	
		1	0.759	0.613	-0.442	1.961	
		2	50.846	0.800	49.278	52.415	
	2	-1	2.260	2.305	-2.257	6.778	
		1	1.570	1.183	-0.748	3.888	
		2	54.307	0.817	52.706	55.909	
Sound environment appropriate:	2	Reference					0.315
	0	-1	-10.618	NaN	NaN	NaN	
		1	-37.181	0.000	-37.181	-37.181	
		2	-0.854	0.000	-0.854	-0.854	
	1	-1	0.405	0.000	0.405	0.405	
		1	60.549	0.000	60.549	60.549	
		2	1.419	0.000	1.419	1.419	
	3	-1	-1.702	1.101	-3.860	0.457	
		1	-0.831	0.629	-2.063	0.401	
		2	13.165	0.723	11.748	14.581	
	4	-1	-3.332	2.174	-7.593	0.928	
		1	-0.691	0.918	-2.491	1.109	
		2	11.233	0.793	9.678	12.788	
AIC	278.4						
McFadden pseudo R^2	0.180						

Table D13 Model 6 in Park 2 (questions related to thermal, visual, and acoustic domain)

Variable		Overall comfort level	Coefficient	Standard error	Confidence intervals 2.5%	97.5%	*p* value
(Intercept)		-1	-43.893	0.000	-43.893	-43.893	
		1	-2.305	4.291	-10.716	6.106	
		2	-211.412	0.966	-213.306	-209.518	
Thermal sensation:	0	Reference					0.328
	1	-1	51.571	0.000	51.571	51.571	
		1	0.276	1.388	-2.444	2.995	
		2	29.079	2.069	25.023	33.134	
	2	-1	90.676	0.000	90.676	90.676	
		1	75.596	NaN	NaN	NaN	
		2	5.476	0.000	5.476	5.476	
Thermal comfort:	0	Reference					0.005
	-1	-1	20.018	0.000	20.018	20.018	
		1	2.159	2.027	-1.814	6.132	
		2	14.269	0.000	14.269	14.269	
	1	-1	-73.762	0.000	-73.762	-73.762	
		1	4.191	1.801	0.661	7.721	
		2	3.973	3.381	-2.653	10.598	
	2	-1	7.635	NaN	NaN	NaN	
		1	37.673	0.000	37.673	37.673	
		2	131.702	0.000	131.702	131.702	
Thermal preference:	0	Reference					0.299
	-1	-1	47.345	0.000	47.345	47.345	
		1	-2.334	1.847	-5.955	1.286	
		2	-5.251	2.688	-10.519	0.018	
	1	-1	58.126	NaN	NaN	NaN	
		1	-5.657	3.114	-11.760	0.447	
		2	-123.160	0.000	-123.160	-123.160	
Thermal acceptability:	0	Reference					0.223
	-2	-1	19.512	NaN	NaN	NaN	
		1	-124.179	0.000	-124.179	-124.179	
		2	4.550	0.000	4.550	4.550	
	-1	-1	32.313	NaN	NaN	NaN	
		1	-109.456	0.000	-109.456	-109.456	
		2	12.288	0.000	12.288	12.288	
	1	-1	73.818	0.000	73.818	73.818	
		1	3.111	1.851	-0.516	6.739	
		2	43.857	1.557	40.805	46.909	
	2	-1	81.277	NaN	NaN	NaN	
		1	-0.777	2.144	-4.980	3.426	
		2	37.179	1.666	33.913	40.445	
Visual sensation:	0	Reference					0.977
	-1	-1	59.836	NaN	NaN	NaN	
		1	69.500	0.000	69.500	69.500	
		2	10.673	0.000	10.673	10.673	
	1	-1	-30.424	0.000	-30.424	-30.424	
		1	1.136	1.791	-2.375	4.647	
		2	26.068	1.856	22.430	29.706	
	2	-1	-74.724	NaN	NaN	NaN	
		1	0.050	2.738	-5.317	5.416	
		2	21.923	1.865	18.268	25.579	
Visual comfort:	0	Reference					0.265
	-1	-1	-61.790	NaN	NaN	NaN	
		1	-7.122	0.000	-7.122	-7.122	
		2	0.325	0.000	0.325	0.325	

	1	-1	-31.960	0.000	-31.960	-31.960	
		1	1.617	1.812	-1.935	5.169	
		2	81.970	1.691	78.657	85.284	
	2	-1	-10.522	NaN	NaN	NaN	
		1	4.150	3.065	-1.858	10.157	
		2	115.505	1.955	111.673	119.337	
Visual preference:	0	Reference					0.982
	-1	-1	-26.113	NaN	NaN	NaN	
		1	-1.329	2.714	-6.649	3.991	
		2	-32.078	0.000	-32.078	-32.078	
	1	-1	-25.130	0.000	-25.130	-25.130	
		1	99.314	0.000	99.314	99.314	
		2	10.252	0.000	10.252	10.252	
Visual acceptability:	0	Reference					0.991
	-1	-1	17.009	0.000	17.009	17.009	
		1	-11.010	0.000	-11.010	-11.010	
		2	4.966	0.000	4.966	4.966	
	1	-1	-76.322	0.000	-76.322	-76.322	
		1	0.356	4.100	-7.680	8.391	
		2	-50.209	2.336	-54.787	-45.630	
	2	-1	-84.388	0.000	-84.388	-84.388	
		1	2.708	4.171	-5.468	10.883	
		2	-50.624	1.873	-54.295	-46.952	
Noise perception:	0	Reference					0.953
	-2	-1	0.700	NaN	NaN	NaN	
		1	59.876	0.000	59.876	59.876	
		2	-9.071	0.000	-9.071	-9.071	
	-1	-1	43.748	0.000	43.748	43.748	
		1	-1.464	1.836	-5.063	2.135	
		2	26.649	1.743	23.233	30.065	
	1	-1	-21.647	0.000	-21.647	-21.647	
		1	-1.084	1.562	-4.146	1.977	
		2	24.141	2.968	18.323	29.959	
	2	-1	-5.868	0.000	-5.868	-5.868	
		1	23.444	0.000	23.444	23.444	
		2	6.957	0.000	6.957	6.957	
Acoustic quality perception:	0	Reference					0.748
	-2	-1	-11.351	0.000	-11.351	-11.351	
		1	28.642	0.000	28.642	28.642	
		2	4.251	0.000	4.251	4.251	
	-1	-1	-48.294	NaN	NaN	NaN	
		1	-71.998	0.000	-71.998	-71.998	
		2	-15.107	0.000	-15.107	-15.107	
	1	-1	-12.295	0.000	-12.295	-12.295	
		1	1.392	1.608	-1.760	4.544	
		2	89.345	0.970	87.443	91.247	
	2	-1	67.211	NaN	NaN	NaN	
		1	2.227	2.860	-3.378	7.832	
		2	109.843	1.153	107.583	112.103	
Sound environment appropriate:	2	Reference					0.851
	0	-1	-31.012	NaN	NaN	NaN	
		1	-6.116	0.000	-6.116	-6.116	
		2	1.423	0.000	1.423	1.423	
	1	-1	15.947	NaN	NaN	NaN	
		1	102.035	0.000	102.035	102.035	
		2	-15.766	0.000	-15.766	-15.766	

	3	-1	0.215	0.000	0.215	0.215
		1	-3.026	1.495	-5.957	-0.095
		2	-29.531	0.970	-31.433	-27.629
	4	-1	-80.956	NaN	NaN	NaN
		1	-2.064	2.466	-6.897	2.769
		2	-49.026	1.153	-51.286	-46.765
AIC	257.5					
McFadden pseudo R^2	0.806					
